# An artesunate-modified half-sandwich iridium(iii) complex inhibits colon cancer cell proliferation and metastasis through the STAT3 pathway†

**DOI:** 10.1039/d4cb00114a

**Published:** 2024-12-17

**Authors:** Dongping Deng, Na Xu, Mengmeng Wang, Guandong Zhang, Yan Su, Hongbao Fang, Zhi Su

**Affiliations:** a Jiangsu Collaborative Innovation Center of Biomedical Functional Materials/Nanjing Drum Tower Hospital, College of Chemistry and Materials Science, Nanjing Normal University Nanjing 210023 China zhisu@njnu.edu.cn suyanahnu@163.com fanghb@njnu.edu.cn; b Department of Rheumatology and Immunology, Jinling Hospital, Affiliated Hospital of Medical School, Nanjing University Nanjing 210002 China; c State Key Laboratory of Coordination Chemistry, School of Chemistry and Chemical Engineering, Chemistry and Biomedicine Innovation Center (ChemBIC), Nanjing University Nanjing 210023 China; d College of Life Science and Chemistry, Jiangsu Key Laboratory of Biological Functional Molecules, Jiangsu Second Normal University Nanjing 210013 China

## Abstract

Colon cancer is one of the most commonly diagnosed cancers and is recognized as the most aggressive tumor of the digestive system. Aberrant activation of signal transducer and activator of transcription 3 (STAT3) is associated with proliferation, metastasis and immunosuppression of the tumor cells. Here, to inhibit the STAT3 pathway and suppress metastasis in colon cancer cells, the half-sandwich iridium complex Ir-ART containing an artesunate-derived ligand was synthesized. The complex showed remarkable antiproliferative activity against human colon cancer HCT-116 cells and exhibited a concentration-dependent reduction in STAT3 protein expression. Mechanism study demonstrates that Ir-ART is located mainly in the nucleus and mitochondria, causing γ-H2AX and cyclin B1 reduction and reactive oxygen species accumulation and mitochondrial membrane potential loss, ultimately leading to autophagic cell death. The migration of cancer cells was also inhibited *via* metalloproteinase 9 downregulation. Furthermore, Ir-ART could initiate antitumor immune responses by eliciting immunogenic cell death and downregulating immunosuppressive cytokine cyclooxygenase-2. Taken together, Ir-ART is expected to be further applied to chemotherapy and immunotherapy for colon cancer.

## Abstract 2

An artesunate-modified half-sandwich iridium(iii) complex inhibits colon cancer cell proliferation and metastasis through the STAT3 pathway.

## Graphical abstract

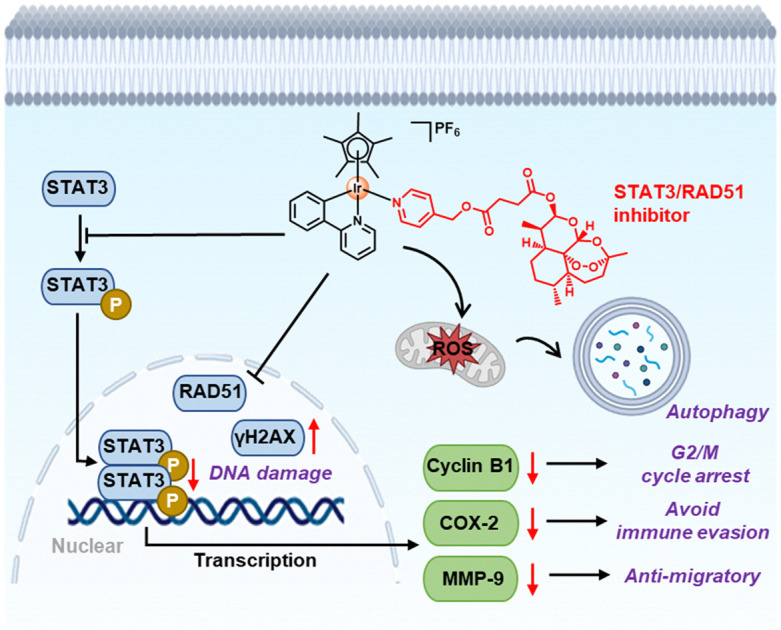

## Introduction

Colon cancer is a relatively common malignant tumor of the gastrointestinal tract, with a high incidence rate among all malignant tumors, and is recognized as the most aggressive tumor in the gastrointestinal system.^1,2^ Signal transducer and activator of transcription 3 (STAT3), a member of the cytoplasmic transcription factor family, transduces extracellular growth factor and cytokine signals and regulates the expression of genes involved in cell proliferation (*e.g.*, cyclin B1), invasion/migration (*e.g.*, metalloproteinases 9), and immune response (*e.g.*, cyclooxygenase-2).^3–6^ Abnormal STAT3 signaling is commonly related to the occurrence and development of colon cancer, and this makes STAT3 targeting an effective strategy for the treatment of colon cancer.^7–9^

Organometallic complexes such as iridium(iii), ruthenium(ii), and platinum(ii) complexes have been widely reported as antitumor drugs due to their particular redox potentials, adjustability of ligand functionalization, and diverse antitumor mechanisms of action.^10–12^ In recent years, a number of metal-based complexes have been found to inhibit tumor proliferation and metastasis *via* combating with STAT3, such as Pt(ii),^13^ Pt(iv),^14^ Rh(iii),^15^ and Ir(iii).^16^ Our group previously designed pterostilbene-derived cyclometalated Ir(iii) complexes as anti-breast cancer and anti-metastasis agents by STAT3 inhibition.^17^ However, the relationship between iridium-based STAT3 inhibition and tumor immunogenicity has not been further investigated. Half-sandwich iridium(iii) complexes, with the common formula of [(Cp*)Ir(L^L′)Z]^0/*n*+^ (Cp*, cyclopentadienyl; L^L′, chelated ligands; Z, leaving groups), have drawn much attention owing to their noticeable biological features and novel capacity in disturbing intracellular redox balance.^18^ The typical (Cp*)Ir(ppy)py (ppy, 2-phenylpyridine; py, pyridine) could cause increased reactive oxygen species (ROS) and depolarization of mitochondrial membrane potential (MMP), resulting in high anticancer activity.^19^ However, the potential of Ir–Cp* complexes as STAT3 inhibitors and immunogenic inducers has not been explored.

Artemisinin is a sesquiterpene lactone compound exacted from the Chinese herb *qinghaosu* and is used as an antimalarial drug.^20^ In the last few years, artemisinin and its derivatives have also been found to have a variety of pharmacological effects, including immunosuppression, antischistosomiasis, antiviral, and antitumor.^21^ Artesunate (ART) was developed as a hemisuccinate derivative of artemisinin and the ART-mediated cytotoxicity has been attributed to ROS generation, cell cycle arrest, and STAT3 signaling pathway inhibition.^22–24^ The Wang group linked ART moieties to the Pt center of oxaliplatin to obtain Pt(iv) complex OPA, which exerted strong inhibition on the expression of triggering receptor expressed on myeloid cells-2 (TREM2) on macrophages and suppresses tumors by remodeling the immunosuppressive microenvironment.^25^ The Li group synthesized two ART conjugated phosphorescent rhenium(i) complexes that induce both apoptosis and ferroptosis in HeLa cells through mitochondrial damage and lipid peroxidation accumulation.^26^ The Ye group designed six ART conjugated Ru(ii) complexes that induce autophagy-dependent cell apoptosis *via* mitochondrial dysfunction and ROS accumulation.^27^ Until now, no ART-decorated metal-based STAT3 inhibitor has been reported, and we proposed that the conjugation between half-sandwich iridium complexes and ART could promote anticancer cytotoxicity and activation of the immune response *via* the STAT3 signaling pathway.

Herein, we designed and synthesized a half-sandwich iridium complex [(Cp*)Ir(ppy)(py-ART)]PF_6_ (Ir-ART) with an ART moiety to raise anticancer activity and activate the immune response by STAT3 inhibition. Due to the high expression of STAT3 in colon cancer, Ir-ART demonstrates the best anti-proliferation ability in HCT-116 colon cancer cells. Ir-ART is located mainly in the nucleus and mitochondria, causing γ-H2AX and cyclin B1 reduction and ROS accumulation and MMP loss, ultimately leading to autophagic cell death. The migration of cancer cells was also inhibited *via* metalloproteinase 9 downregulation. Furthermore, Ir-ART could initiate antitumor immune responses by eliciting immunogenic cell death and downregulating immunosuppressive cytokine cyclooxygenase-2. This study not only provides a novel metal-Cp*-based STAT3 inhibitor chemoimmunotherapeutic agents for treating colon cancer, but also further highlights the efficacy of the metal–ligand synergetic enhancement strategy in cancer treatment.

## Results and discussion

### Synthesis and characterization

**Fig. 1:**
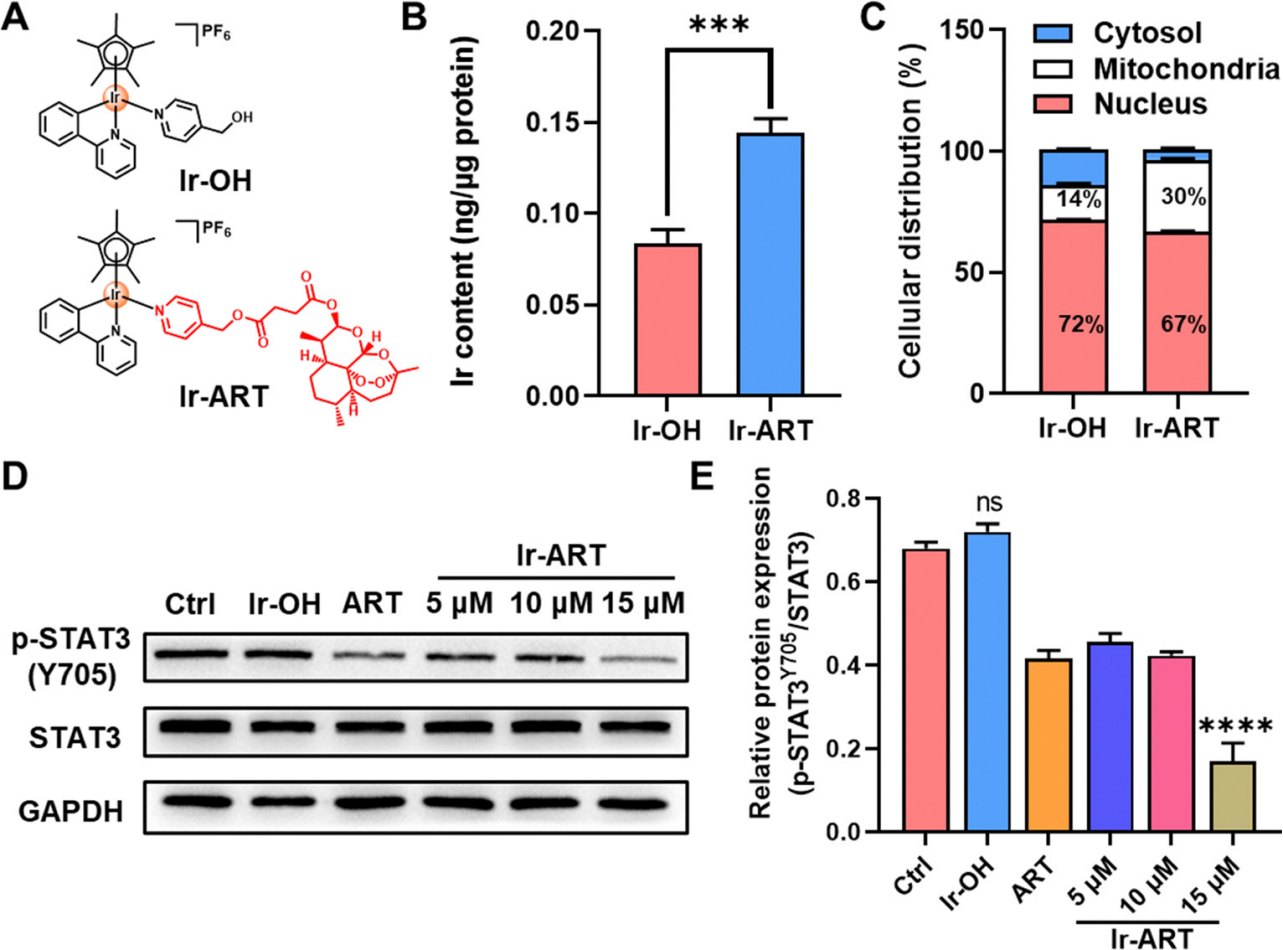
Chemical structure and STAT3 inhibition of Ir-ART. (A) Chemical structure of Ir-OH and Ir-ART. (B) Ir accumulation (ng/μg protein) and (C) cellular distribution in HCT-116 cells after treatment with Ir-OH and Ir-ART (10 μM), respectively, for 6 h as measured by ICP-MS. (D) Western blot and (E) corresponding quantitative analysis of p-STAT3 and STAT3 in HCT-116 cells after incubation with Ir-OH (15 μM), ART (15 μM), and Ir-ART (5, 10, 15 μM), respectively, for 24 h.

The modified py-ART ligand was synthesized from pyridin-4-ylmethanol with artesunate (ART). Complex [(Cp*)Ir(ppy)(py-ART)]PF_6_ (Ir-ART, Cp* = pentamethylcyclopentadienyl, ppy = 2-phenylpyridine) was prepared according to the previous literature methods with minor modifications,^28^ and complex Ir-OH ([(Cp*)Ir(ppy)(py-OH)]PF_6_, py-OH = 4-pyridylcarbinol) without modified ART ligand was also synthesized as a control (Fig. 1A and Scheme S1, ESI†). All complexes were fully characterized with ^1^H and ^13^C nuclear magnetic resonance (NMR) and electrospray ionization-mass spectrometry (ESI-MS) (Fig. S1–S9, ESI†).

Ester-modified compounds have been reported to be hydrolyzed by a variety of esterases,^29^ and we first investigated the stability of Ir-ART in esterase solutions using porcine liver esterase (PLE) as a model. No corresponding hydrolysis peaks were observed in ESI-MS after incubation of PLE with Ir-ART for 24 h (Fig. S10, ESI†), indicating that Ir-ART has good stability, which is sufficient for further biological studies and mechanism exploration. Besides, we evaluated the stability of Ir-ART under cellular(-like) conditions, which was detected in a 1% DMSO/99% DMEM (v/v) solution by UV-visible spectrophotometry at 12 h intervals (Fig. S11, ESI†). The spectra of Ir-ART changed only slightly within 24 h, indicating that it is sufficiently stable under cellular(-like) conditions.

### Cytotoxic *in vitro* experiments

The antiproliferative activity of the compounds was evaluated by the MTT assay on several human cancer cell lines, including HCT-116 (colon cancer), A549 (lung cancer), MCF-7 (breast cancer), and HeLa (cervical cancer). The half maximal inhibitory concentrations (IC_50_) of the compounds towards these cell lines at 48 h are listed in Table 1. Free ART was almost nontoxic below 36 μM, which may be due to the poor water solubility and low bioavailability of natural products,^30^ and Ir-OH manifested moderate cytotoxicity against the tested cancer cells (IC_50_ values, 20–47 μM). Due to the high expression of STAT3 protein in HCT-116 cells compared to other cell lines, complex Ir-ART showed the best cytotoxicity against HCT-116 cells, with an IC_50_ value of 6.4 μM, which is almost similar to cisplatin. This demonstrated that the introduction of ART into a half-sandwich iridium complex was an effective strategy to combat colon cancer cells.

**Table 1:** The IC_50_ (μM) values for ART, Ir-OH, Ir-ART, and cisplatin against cancer cell lines for 48 h. Data are presented as the mean ± SD (standard error of the mean) from three independent experiments

Compounds	HCT-116	A549	HeLa	MCF-7
ART	36.4 ± 2.3	75.3 ± 1.4	67.9 ± 2.7	>100
Ir-OH	22.6 ± 0.9	47.7 ± 0.9	39.4 ± 1.5	25.6 ± 0.7
Ir-ART	6.4 ± 0.1	21.4 ± 0.6	24.7 ± 0.8	16.6 ± 1.4
Cisplatin	5.3 ± 0.4	7.7 ± 0.5	11.0 ± 0.3	5.9 ± 0.1

### Cellular uptake and STAT3 inhibition

Drug cellular uptake and cytotoxicity were usually associated with lipophilicity (log *P*_o/w_), with the decoration of ART, the log *P*_o/w_ of Ir-ART was elevated to 2.07, compared to 0.97 of Ir-OH (Fig. S12, ESI†), suggesting that Ir-ART may be easier to enter cells than Ir-OH and would benefit its bio-application. To further determine cellular uptake, the Ir content of Ir-ART and Ir-OH in HCT-116 after 6 h incubation was quantitatively determined by inductively coupled plasma mass spectrometry (ICP-MS). The cellular Ir content of Ir-ART in HCT-116 was 2.1-fold higher than that of Ir-OH, suggesting that the introduction of ART with metallodrugs greatly enhanced the cellular uptake of Ir-ART (Fig. 1B). Moreover, the intracellular distribution of Ir-ART was evaluated. The nuclear targeting capacity of Ir-ART was maintained, as 67% of the total Ir content accumulated in the nucleus of HCT-116 cells, and the mitochondrial targeting ability was enhanced, possibly due to the ability of ART to target mitochondria (Fig. 1C).^31^

Considering the suppression of the STAT3 pathway by ART,^32^ the STAT3 inhibitory activity in the Ir-ART treated HCT-116 cells was examined. Tyrosine residue phosphorylation (Y705) mainly mediates the nuclear transcriptional function of STAT3, thereby activating various aberrant oncogenes.^33^ The level of phosphorylated STAT3 (p-STAT3 Y705) was determined using western blot analysis. As shown in Fig. 1D and E, the expression of p-STAT3 in HCT-116 cells was suppressed in the presence of Ir-ART, while the expression of STAT3 was almost concordant, which was consistent with previous studies. The above results indicated that Ir-ART could effectively inhibit the activation of STAT3.

### DNA damage and cell cycle arrest

**Fig. 2:**
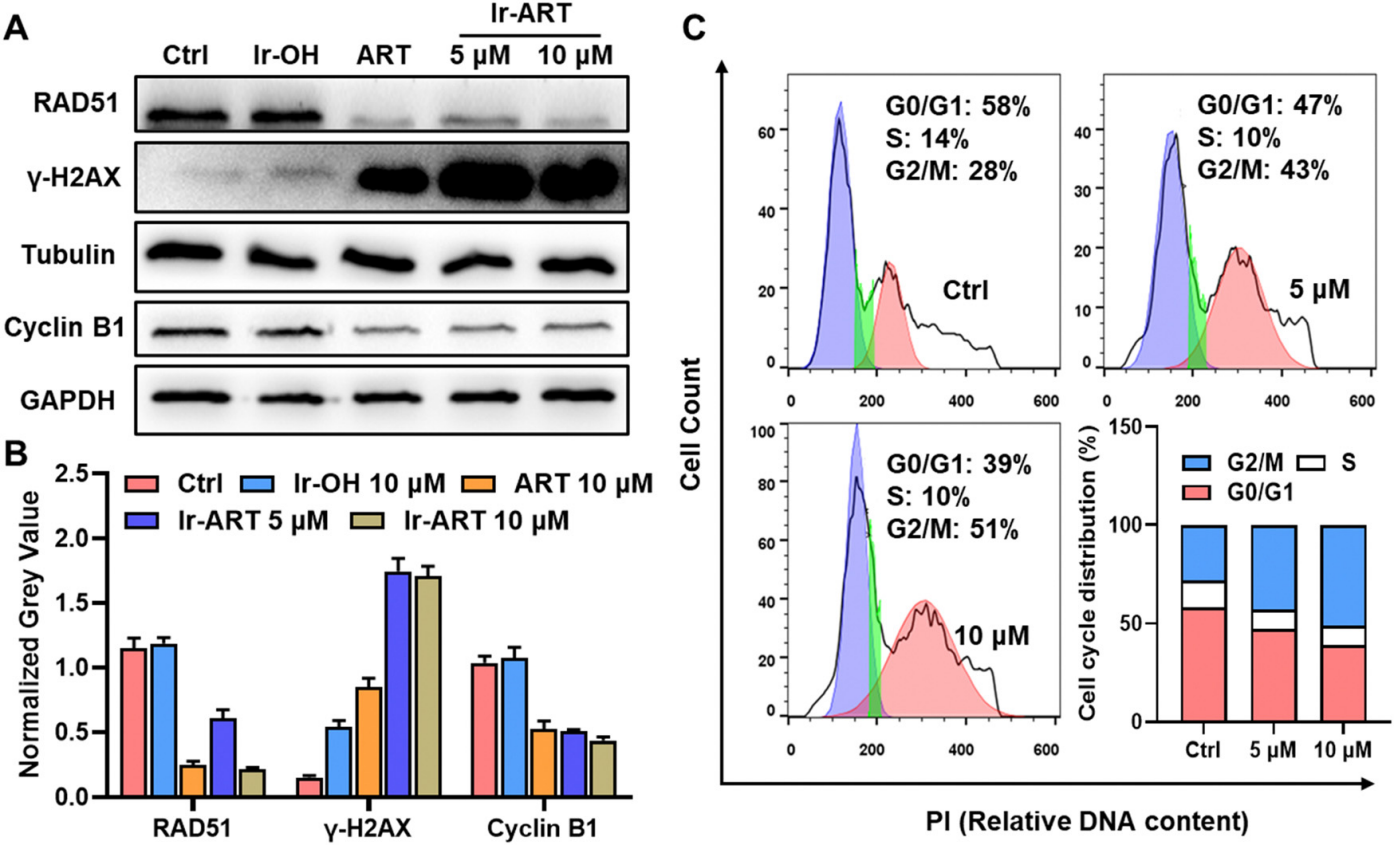
DNA damage and cell cycle arrest induced by Ir-ART. (A) Western blot and (B) corresponding quantitative analysis of RAD51, γ-H2AX and cyclin B1 in HCT-116 cells treated with Ir-OH (10 μM), ART (10 μM), and Ir-ART (5, 10 μM), respectively, for 24 h. (C) Flow cytometry results of cell cycle distribution of HCT-116 cells after incubation with Ir-ART (5, 10 μM) for 24 h. PI: *λ*_ex_ = 488 nm, *λ*_em_ = 580 ± 20 nm.

RAD51 has been shown to be a key DNA damage repair protein, and its overexpression not only promotes cancer progression but also enables cancer cells to resist DNA damage agents.^34^ Previous studies reported that ART could effectively down-regulate RAD51 in cancer cells.^35^ Since 64% of the total Ir-ART localized in the nuclei of HCT-116 cells, the expression of γ-H2AX (a biomarker of DNA double strand breaks) and RAD51 was determined by western blot analysis. As displayed in Fig. 2A and B, the expression of RAD51 was inhibited, and the expression of γ-H2AX was dramatically upregulated in a concentration dependent manner in the Ir-ART treated HCT-116 cells, suggesting that Ir-ART could induce severe DNA damage by downregulating DNA damage repair protein RAD51.

Cellular DNA damage could induce cell cycle arrest, which was examined in HCT-116 cells by flow cytometry after treatment with Ir-ART. Flow cytometric analysis indicated that the cell cycle for the Ir-ART treated HCT-116 cells could be arrested in the G2/M phase in a concentration dependent manner, with the population increasing from 28% to 51% (Fig. 2C). The G2/M cell cycle arrest induced by Ir-ART was also confirmed by the downregulation of cell cycle checkpoint protein cyclin B1 (Fig. 2A), which was regulated by STAT3. These results suggest that Ir-ART could cause cell cycle arrest by decreasing the expression of cyclin B1.

### Mitochondrial dysfunction

**Fig. 3:**
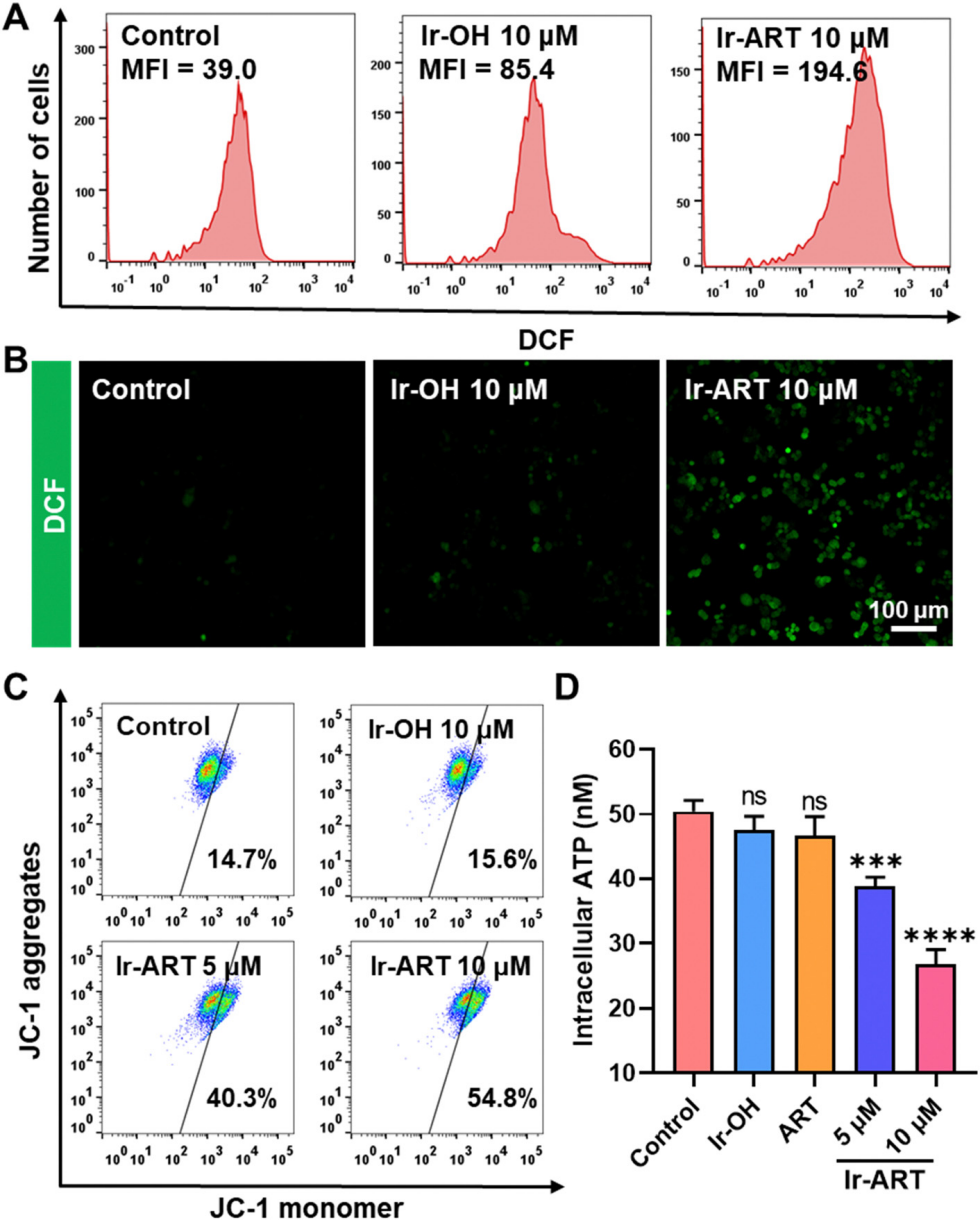
The mitochondrial dysfunction induced by Ir-ART in HCT-116 cells. (A) Flow cytometric analysis and (B) confocal imaging of ROS generation in HCT-116 cells stained with the DCFH-DA probe after incubation with Ir-OH (10 μM) and Ir-ART(10 μM), respectively for 24 h. DCFH-DA: *λ*_ex_ = 488 nm, *λ*_em_ = 520 ± 20 nm. (C) Flow cytometry quantification of JC-1-labeled HCT-116 cells after 24 h treatment with Ir-OH (10 μM) and Ir-ART (5, 10 μM), respectively, for 24 h. Monomer: *λ*_ex_ = 488 nm, *λ*_em_ = 520 ± 20 nm; aggregates: *λ*_ex_ = 488 nm, *λ*_em_ = 580 ± 20 nm. (D) Analysis of intracellular ATP levels in HCT-116 cells after treatment with Ir-OH (10 μM), ART (10 μM), and Ir-ART (5, 10 μM), respectively, for 24 h.

Since Ir-ART was able to partially target mitochondria in HCT-116 cells, as 30% of the total Ir content accumulated in the mitochondria, the effect on mitochondrial function was investigated. An endoperoxide bridge in ART was known to induce intracellular production of highly toxic reactive oxygen species (ROS).^36^ To assess the intracellular ROS levels, HCT-116 cells were treated with different drugs for 24 hours and assayed by flow cytometry utilizing the DCFH-DA probe. As shown in Fig. 3A, cells treated with Ir-ART showed a higher mean fluorescence intensity (MFI = 194.6) than the control (MFI = 39.0) and Ir-OH (MFI = 85.4) groups, suggesting that elevated ROS levels might be closely related to the ART fraction. In addition, confocal laser scanning microscopy (CLSM) further demonstrated that Ir-ART produced higher ROS than the control and Ir-OH groups (Fig. 3B).

Elevated ROS could lead to mitochondrial damage,^37^ and a JC-1 assay kit was subsequently used to assess the effect on mitochondrial membrane potential (MMP).^38^ As depicted in Fig. 3C, in comparison with the control, a significant dose-dependent loss of MMPs was observed in Ir-ART, dropping from 14.7% to 54.8%, indicating that Ir-ART could result in severe depolarization of MMP. CLSM analysis was also performed to detect the MMP in HCT-116 cells. Ir-ART-treated HCT-116 cells emitted strong green fluorescence (Fig. S13, ESI†), suggesting that Ir-ART has a strong ability to impair the MMP. Furthermore, mitochondria could act as bioenergetic centers for cellular metabolism, thus exploring intracellular adenosine triphosphate (ATP) levels. As shown in Fig. 3D, the intracellular ATP level of HCT-116 cells significantly decreased in a dose-dependent manner after the treatment with Ir-ART. All the above results indicated that mitochondrial function was severely disrupted after Ir-ART treatment.

### Cell death mechanism

**Fig. 4:**
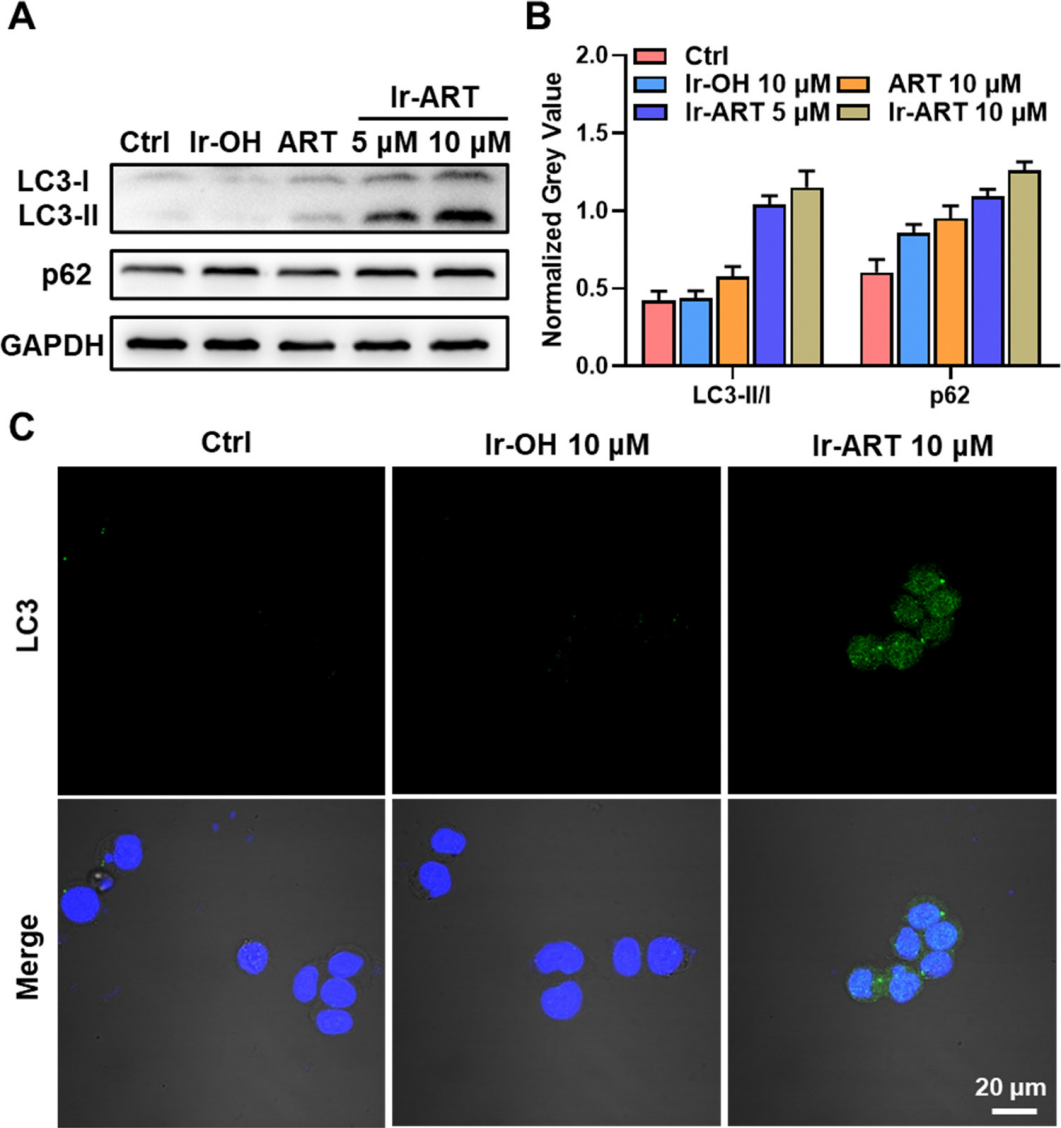
The cell death mode of autophagy after Ir-ART treatment. (A) Immunoblotting of LC3 and p62 in HCT-116 cells treated with Ir-OH (10 μM), ART (10 μM), or Ir-ART (5, 10 μM) for 24 h. (B) Quantitative analysis of immunoblotting in (A). (C) Immunofluorescence assay of LC3 expression in HCT-116 cells treated with Ir-OH (10 μM) and Ir-ART (10 μM) for 24 h. LC3 antibody: *λ*_ex_ = 488 nm, *λ*_em_ = 525 ± 20 nm. DAPI: *λ*_ex_ = 405 nm, *λ*_em_ = 430 ± 20 nm.

Several studies have shown that enhanced ROS and mitochondrial damage could induce cell death through autophagic mechanisms.^39,40^ Autophagy is marked by the conversion of LC3 protein from type I to type II, as shown in Fig. 4A and B, and the ratio of LC3-II to LC3-I was significantly increased after Ir-ART treatment compared with the control group. The activation of autophagy in Ir-ART-treated HCT-116 cells was further demonstrated by immunostaining of LC3B. The dot-like structure of LC3B that emits green fluorescence can be clearly observed in the confocal image (Fig. 4C). Furthermore, Ir-ART was detected to elevate the expression of another autophagy-related protein, SQSTM1/p62 (Fig. 4A and B), which could inhibit the degradation of autophagosomes in cancer cells and favor anti-tumor activity. Taken together, Ir-ART mediated autophagic cell death in HCT-116 colon cancer cells.

### Inhibition of cell migration

**Fig. 5:**
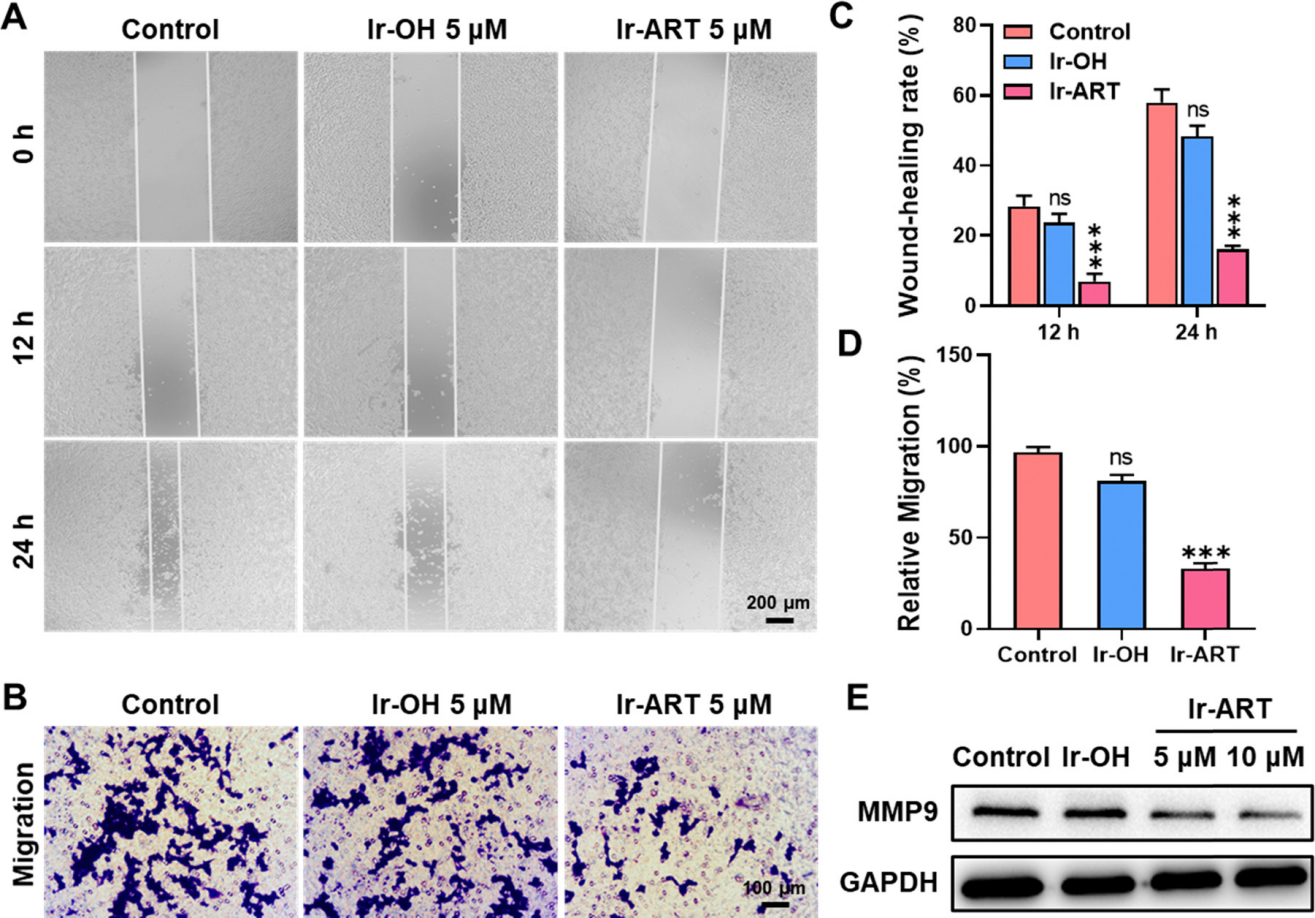
The effects of Ir-ART on cell migration. (A) Representative images of the wound-healing assay in HCT-116 cells after incubation with Ir-OH (5 μM) or Ir-ART (5 μM) for 12 h and 24 h, and (C) the percent of wound-healing closure was calculated. (B) Representative images of HCT-116 cells were observed by transwell migration assay after exposure with Ir-OH (5 μM) or Ir-ART (5 μM) for 24 h, and (D) the number of migrated cells was counted. (E) Immunoblotting of MMP9 in HCT-116 cells treated with Ir-OH (10 μM) or Ir-ART (5, 10 μM) for 24 h.

Migration is a key step in cancer progression, and aberrantly activated STAT3 could promote tumor invasion and metastasis by inducing the expression of matrix metalloproteinases (MMPs, especially MMP-1, MMP-2, and MMP-9).^41^ Therefore, STAT3 was regarded as a potential target for inhibiting cancer metastasis. The scratch wound healing assay and transwell assay were performed to investigate the effect of Ir-ART on cell migration. The concentration of Ir-ART was set to 5 μM, which showed no obvious cytotoxicity during the treatment. As shown in Fig. 5A and C, the wound closure ratio of Ir-ART-treated HCT-116 cells was only 16%, in contrast to 58% of the control group and 48% of the Ir-OH-treated group after 24 h incubation. The transwell assay similarly demonstrated that Ir-ART effectively inhibited cell migration, as shown in Fig. 5B and D, where Ir-ART significantly reduced the number of cells migrating through the wells. Furthermore, the expression of MMP9, a marker of tumor metastasis, was detected by western blotting assay. As shown in Fig. 5E, compared with the control, a decrease in the expression of MMP9 was observed in Ir-ART-treated HCT-116 cells. These results suggested that Ir-ART has the potential to effectively inhibit cancer cell metastasis by suppressing the activation of STAT3-mediated downstream signaling pathways.

### ICD effect and inhibition on immunosuppressive responses by Ir-ART

**Fig. 6:**
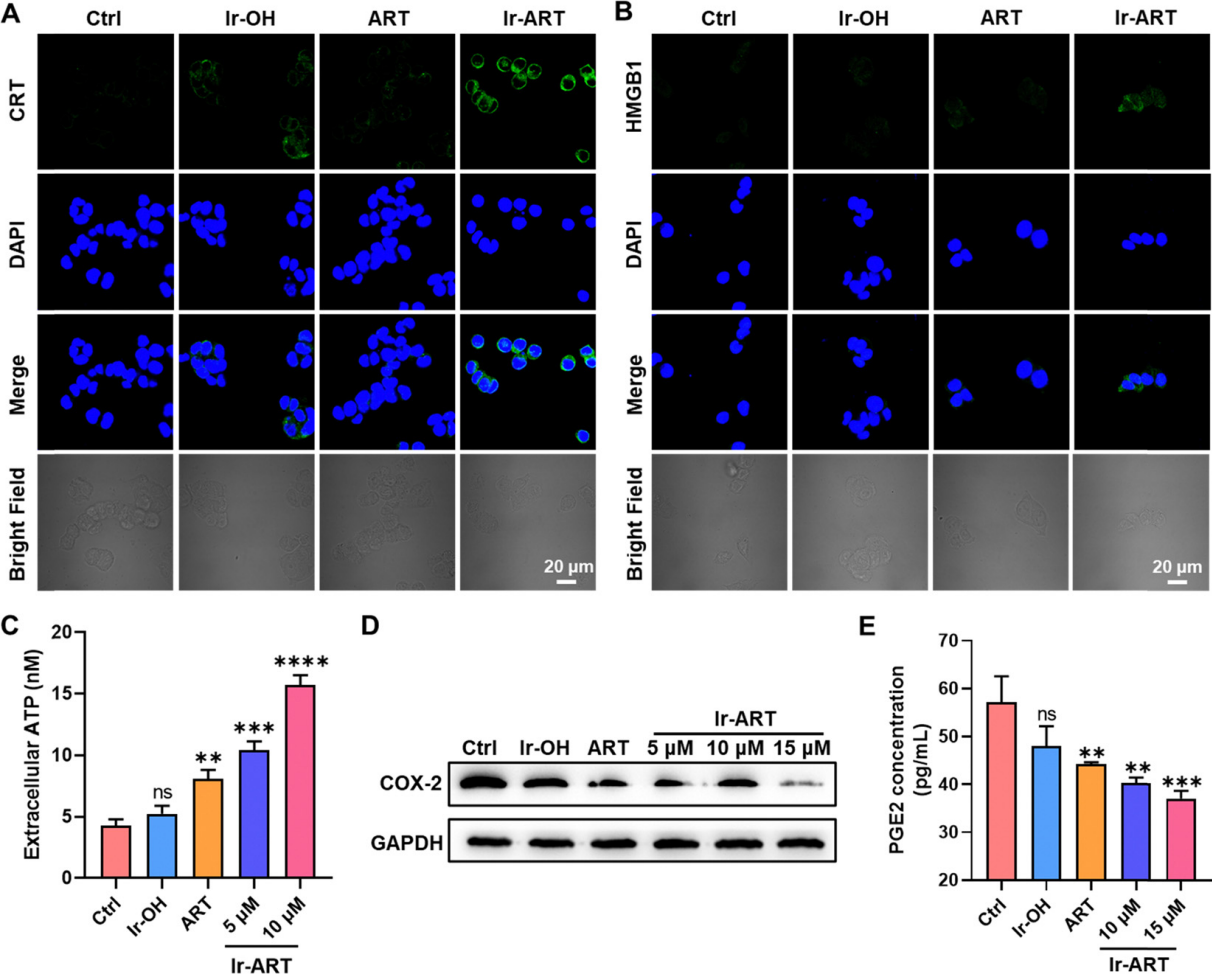
ICD effect and inhibition on immunosuppressive responses in HCT-116 cells treated with Ir-ART. (A) and (B) Confocal images of CRT and HMGB1 release in HCT-116 cells incubated with Ir-OH, ART and Ir-ART (10 μM) for 24 h. CRT antibody: *λ*_ex_ = 488 nm, *λ*_em_ = 525 ± 20 nm. HMGB1 antibody: *λ*_ex_ = 488 nm, *λ*_em_ = 525 ± 20 nm. DAPI: *λ*_ex_ = 405 nm, *λ*_em_ = 430 ± 20 nm. (C) Analysis of ATP levels in HCT-116 cell culture supernatants after treatment with Ir-OH (10 μM), ART (10 μM), or Ir-ART (5, 10 μM) for 24 h. (D) Immunoblotting of COX-2 expression in HCT-116 cells treated with Ir-OH (15 μM), ART (15 μM), or Ir-ART (5, 10, 15 μM) for 24 h. (E) PGE2 concentrations in the extracellular medium supernatant were measured by ELISA.

Inhibition of STAT3 in cancer cells may enhance chemotherapy-associated anticancer immune responses and contribute to the induction of immunogenic cell death (ICD).^42^ In addition, stattic, one of the most classical inhibitors of STAT3, can also increase ICD markers, such as calreticulin (CRT) expression, and high mobility group protein B1 (HMGB1) release in cancer cells.^43^ We wonder if Ir-ART could also induce the ICD effect. Therefore, we investigated the hallmarks of ICD, including the surface-exposure of CRT, the release of HMGB1, and the secretion of ATP. We detected the CRT and HMGB1 by immunofluorescence imaging and flow cytometry. After Ir-ART treatment, we observed the exposure of CRT and the release of HMGB1 (Fig. 6A and B and Fig. S14, ESI†). Meanwhile, extracellular ATP content increased upon Ir-ART treatment (Fig. 6C). These results suggested that Ir-ART can be expected to initiate antitumor immune responses by eliciting ICD. On the other hand, over-activation of STAT3 in cancer cells leads to an increase in the immunosuppressive cytokine cyclooxygenase-2 (COX-2),^44^ and COX-2 is a key rate-limiting enzyme for prostaglandin E2 (PGE2) synthesis,^45^ which can transfer cytokines by regulating antigen-presenting dendritic cells, leading to a decrease in the activation of cytotoxic CD8^+^ T cells and thus allowing tumor cells to escape from immune surveillance.^46^ Therefore, inhibition of COX-2 can effectively avoid immune evasion by tumors. The effect of the complex Ir-ART on COX-2 expression in HCT-116 cells and PGE2 level in the extracellular medium supernatant was determined by western blotting and ELISA, respectively. As shown in Fig. 6D and E, Ir-ART significantly suppressed the level of COX-2 and PGE2 compared to the control. These results collectively indicated that Ir-ART is expected to avoid immune evasion and further amplify antitumor immunity by inhibiting COX-2 and PGE2.

## Conclusions

In this work, we designed and synthesized Ir-ART, a half-sandwich iridium based STAT3 inhibitor, for colon cancer therapy. The results of *in vitro* cytotoxicity assays showed that Ir-ART exhibited high anti-proliferation activity against HCT-116 colon cancer cells. Ir-ART is located in the nucleus and mitochondria, causing severe nuclear damage and mitochondrial dysfunction, ultimately leading to autophagic cell death. Besides, Ir-ART could initiate antitumor immune responses by eliciting ICD. More importantly, we demonstrated that Ir-ART has a strong STAT3 inhibitory activity, which can inhibit the phosphorylation of STAT3 and the expression of related proteins cyclin B1, MMP9, and COX-2, which not only enabled Ir-ART to successfully inhibit the migration of cancer cells, but also amplified the antitumor immune response by remodeling the tumor immunosuppressive microenvironment. In short, we developed a novel metal-Cp*-based STAT3 inhibitor with anti-proliferative and anti-migratory capabilities, which is expected to be further used in tumor chemotherapy and immunotherapy.

## Author contributions

Dongping Deng: writing – original draft, investigation, data curation. Na Xu: methodology, investigation, formal analysis. Guandong Zhang: methodology, investigation, formal analysis. Mengmeng Wang: methodology, investigation. Yan Su: writing – review & editing, supervision, funding acquisition, conceptualization. Hongbao Fang: funding acquisition, supervision. Zhi Su: conceptualization, funding acquisition, supervision, writing – review & editing.

## Data availability

The data supporting this article have been included as part of the ESI.†

## Conflicts of interest

There are no conflicts to declare.

## Supplementary Material

CB-006-D4CB00114A-s001

CB-006-D4CB00114A-s002
