# Supplementary material for: An artesunate-modified half-sandwich iridium(iii) complex inhibits colon cancer cell proliferation and metastasis through the STAT3 pathway
Source: RSC Chem Biol. 2024 Dec 17;6(2):218–26. doi: 10.1039/d4cb00114a (PMC11651070; doi:10.1039/d4cb00114a)
Supplement: CB-006-D4CB00114A-s001 [file CB-006-D4CB00114A-s001.pdf]

This journal is © The Royal Society of Chemistry 2024

Western-blot uncropped images

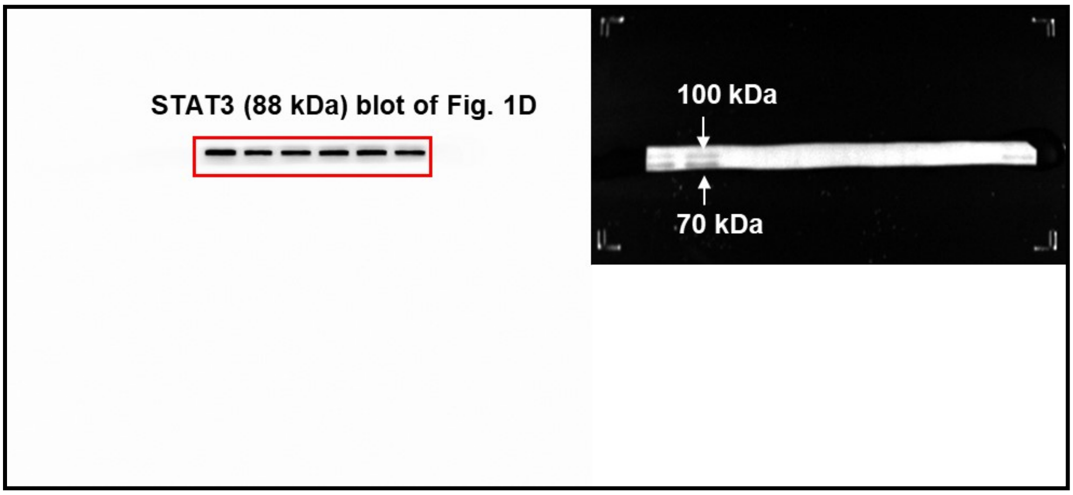

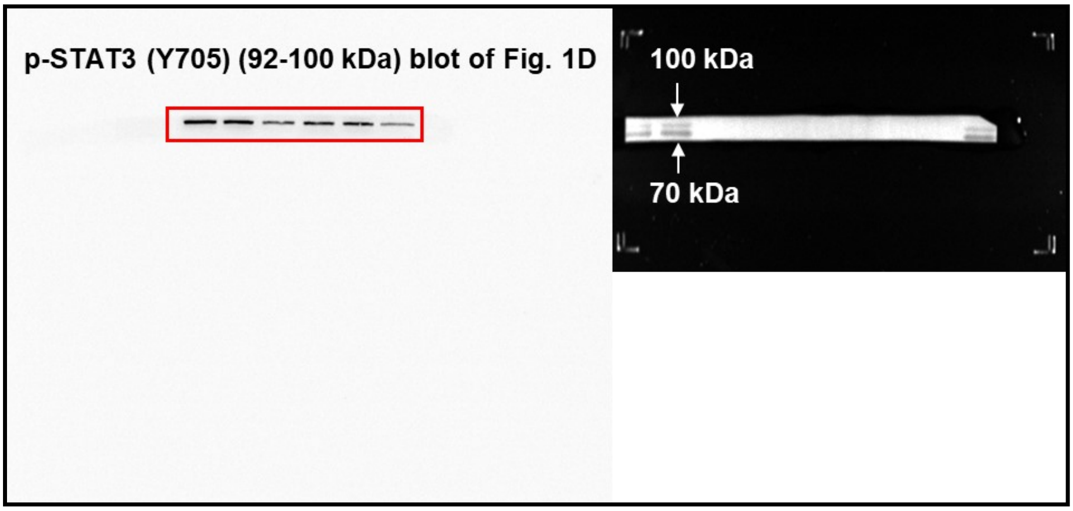

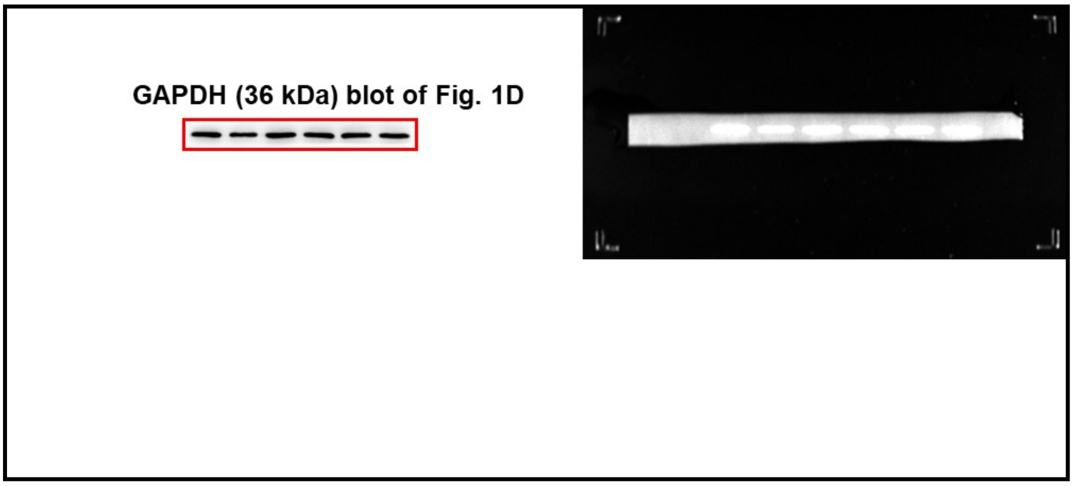

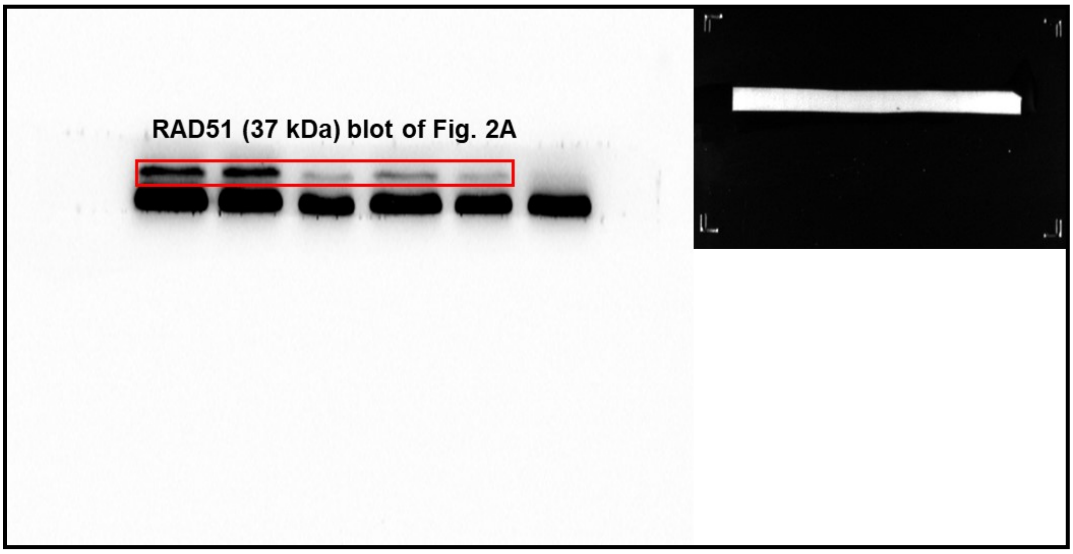
RAD51 (37 kDa) blot of Fig. 2A

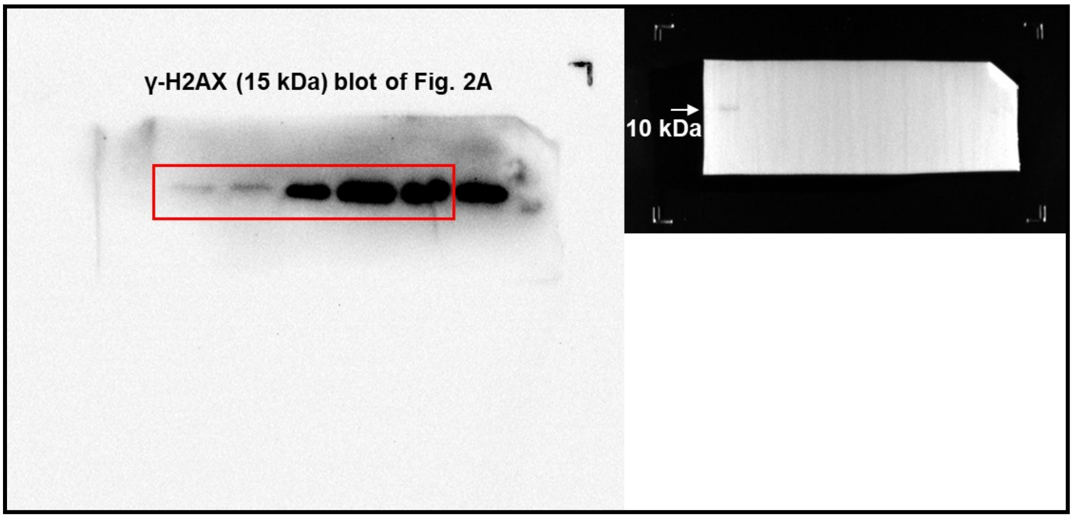
γ-H2AX (15 kDa) blot of Fig. 2A
10 kDa

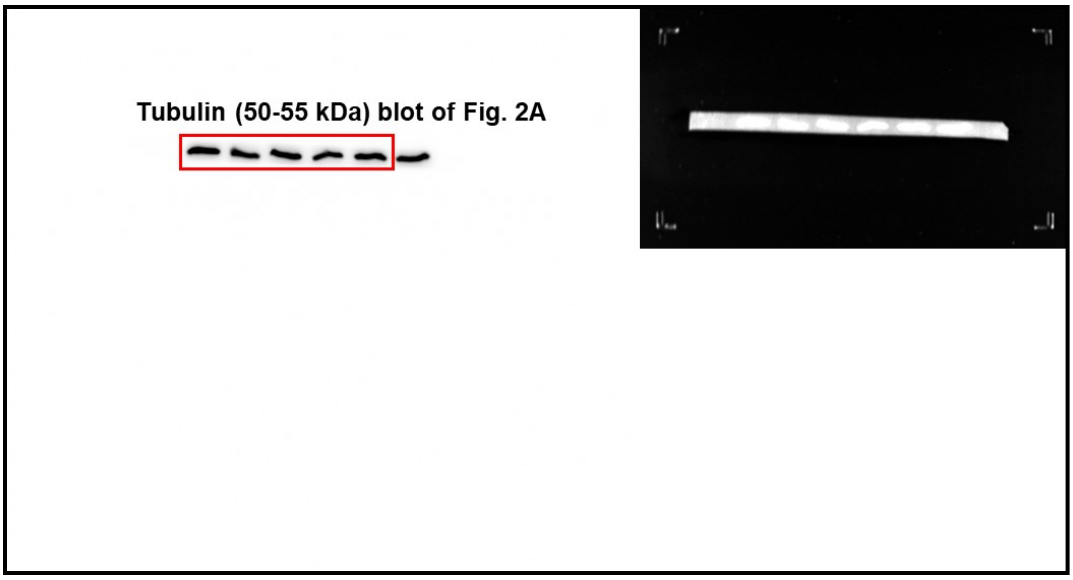
Tubulin (50-55 kDa) blot of Fig. 2A

Cyclin B1 (55-60 kDa) blot of Fig. 2A

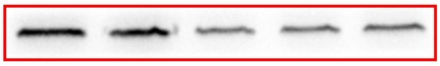

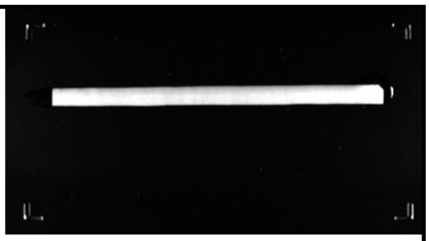

GAPDH (36 kDa) blot of Fig.2A

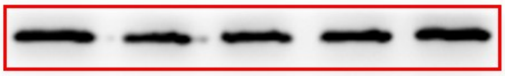

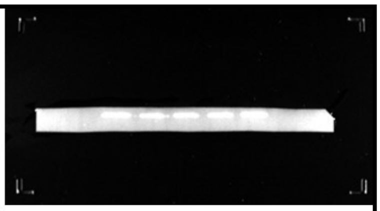

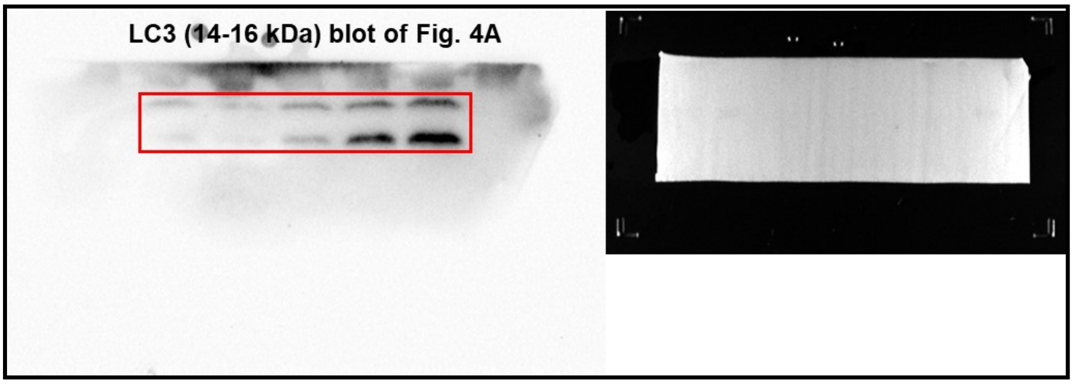
LC3 (14-16 kDa) blot of Fig. 4A

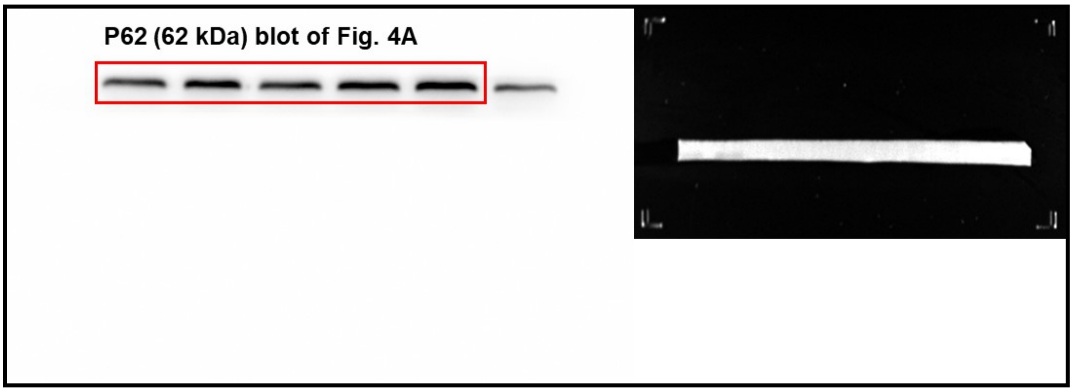
P62 (62 kDa) blot of Fig. 4A

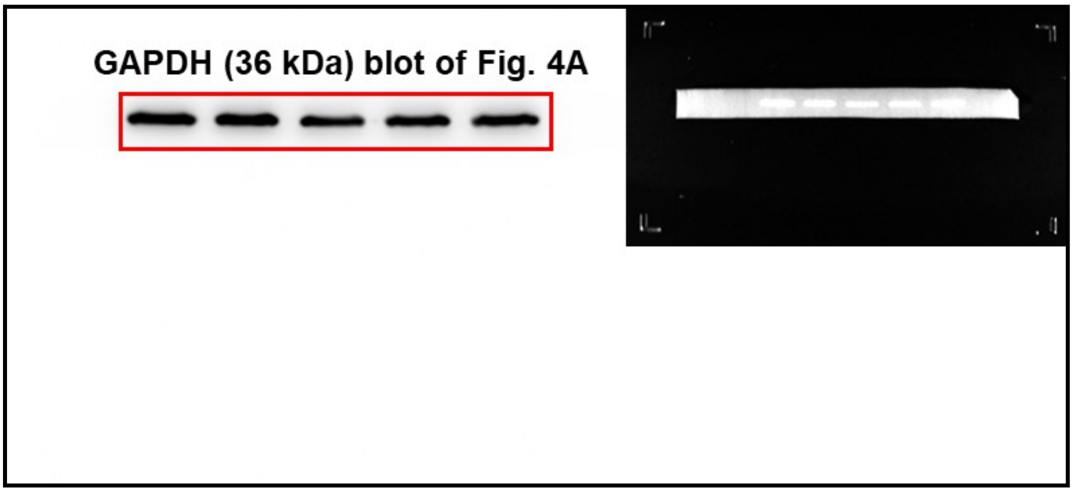
GAPDH (36 kDa) blot of Fig. 4A

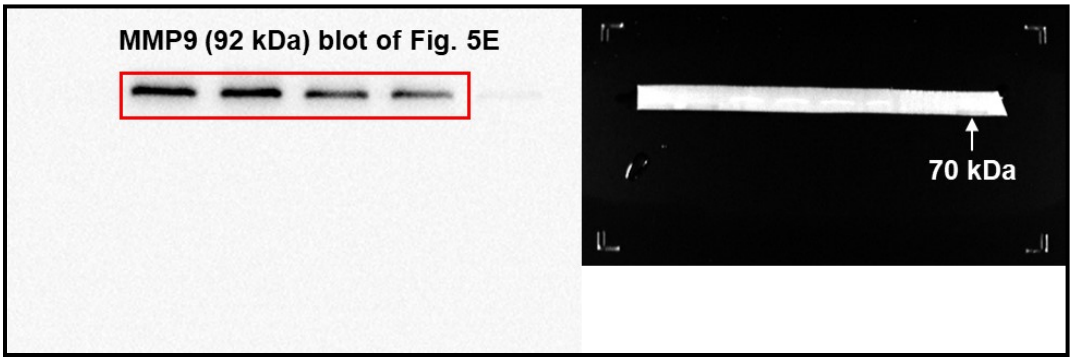
MMP9 (92 kDa) blot of Fig. 5E
70 kDa

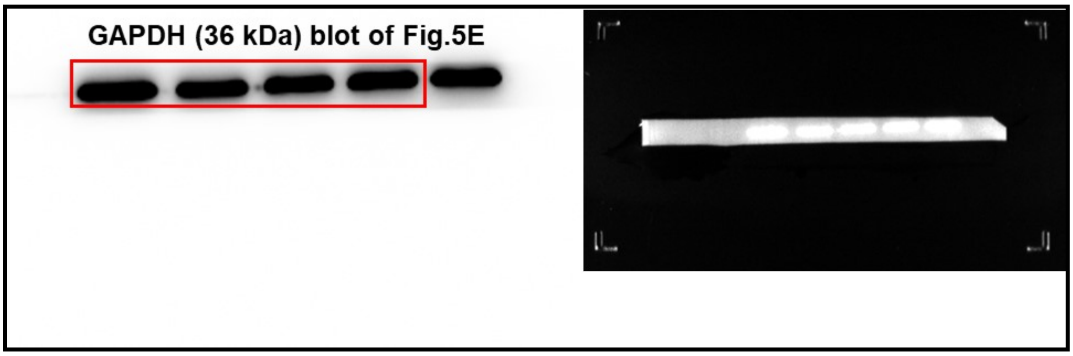
GAPDH (36 kDa) blot of Fig.5E

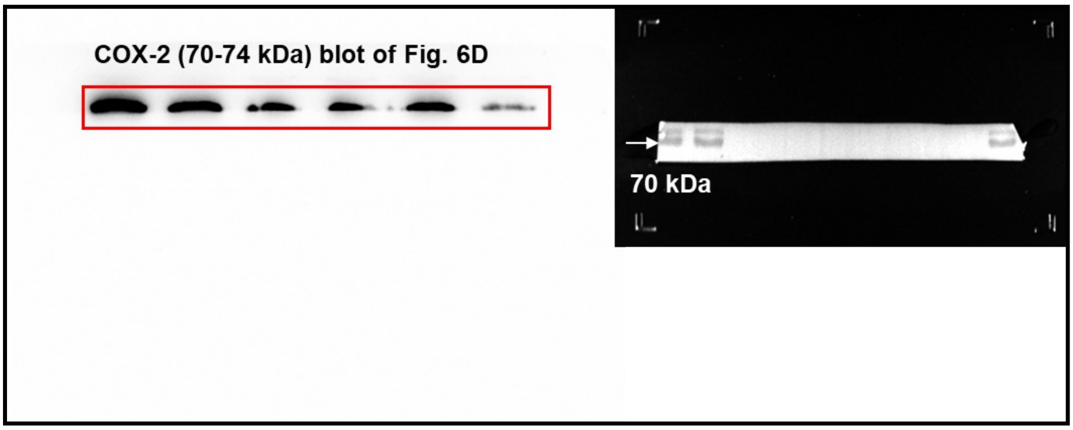
COX-2 (70-74 kDa) blot of Fig. 6D
70 kDa

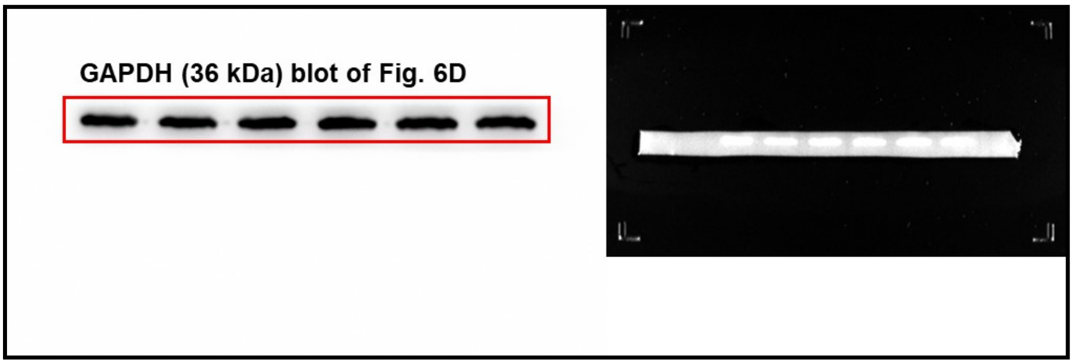
GAPDH (36 kDa) blot of Fig. 6D

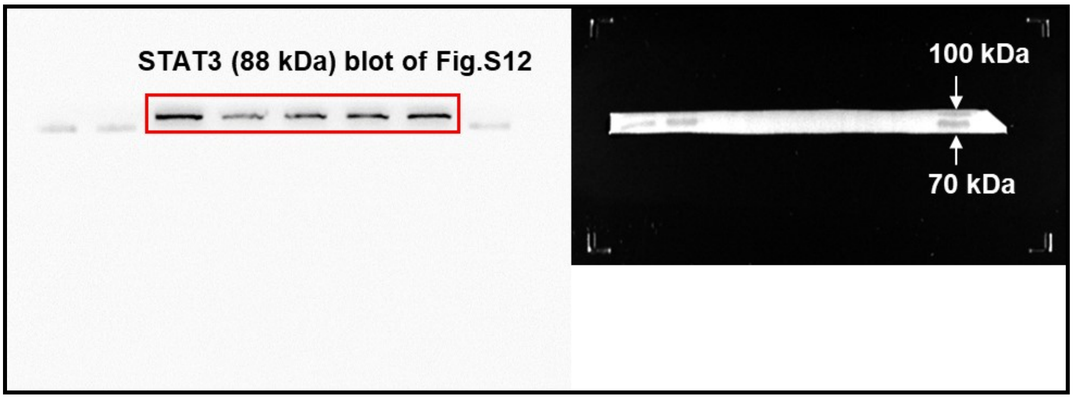
STAT3 (88 kDa) blot of Fig.S12
100 kDa
70 kDa

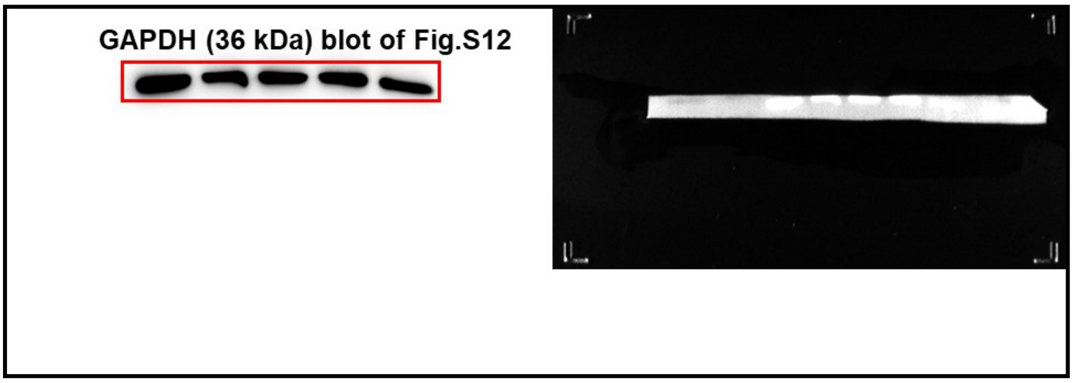
GAPDH (36 kDa) blot of Fig.S12
