# Supplementary material for: An artesunate-modified half-sandwich iridium(iii) complex inhibits colon cancer cell proliferation and metastasis through the STAT3 pathway
Source: RSC Chem Biol. 2024 Dec 17;6(2):218–26. doi: 10.1039/d4cb00114a (PMC11651070; doi:10.1039/d4cb00114a)
Supplement: CB-006-D4CB00114A-s002 [file CB-006-D4CB00114A-s002.pdf]

Supplementary Information (SI) for RSC Chemical Biology.
This journal is © The Royal Society of Chemistry 2024

Supplementary information

Artesunate-modified half-sandwich iridium(III) complex inhibits colon cancer cell proliferation and metastasis through STAT3 pathway

Dongping Deng [a], Na Xu, [a], Mengmeng Wang [a,d], Guandong Zhang, [a] Yan Su [a,b,*], Hongbao Fang [a,c,*], Zhi Su [a,*]

[a] Jiangsu Collaborative Innovation Center of Biomedical Functional Materials/Nanjing Drum Tower Hospital, College of Chemistry and Materials Science, Nanjing Normal University, Nanjing 210023, China;

[b] Department of Rheumatology and Immunology, Jinling Hospital, Affiliated Hospital of Medical School, Nanjing University, Nanjing 210002, China

[c] State Key Laboratory of Coordination Chemistry, School of Chemistry and Chemical Engineering, Chemistry and Biomedicine Innovation Center (ChemBIC), Nanjing University, Nanjing 210023, China.

[d] College of Life Science and Chemistry, Jiangsu Key Laboratory of Biological Functional Molecules, Jiangsu Second Normal University, Nanjing 210013, China

[*] Corresponding authors: zhisu@njnu.edu.cn; suyanahnu@163.com; fanghb@njnu.edu.cn;

Materials and methods

Materials and instrumentation

Artesunate (ART), (pentamethylcyclopentadienyl)iridium(III) chloride dimer, ppy (2-Phenylpyridine), 4-pyridylcarbinol, 1-(3-dimethylaminopropyl)-3-ethylcarbo-diimide hydrochloride (EDCI), trimethylamine (TEA), 4-dimethylaminopyridi (DMAP) (HEOWNS), cisplatin (Energy Chemical), DMSO (dimethyl sulfoxide, Sigma Aldrich), MTT (3-(4,5-dimethylthiazol-2-yl)-2,5-diphenyltetrazolium bromide, HEOWNS), DMEM (Dulbecco's modified Eagle's medium, Gibco BRL) or RPMI 1640 (Roswell Park Memorial Institute medium 1640, Gibco BRL) medium containing 10 % FBS (fetal bovine serum, Gibco BRL), 100 μg/mL streptomycin, and 100 U/mL penicillin (Gibco BRL). ROS assay kit, JC-1 assay kit, cell cycle detection kit, Mitochondria/Nuclei Isolation kit, crystal violet staining solution and BCA Protein Quantitation Assay were purchased from KeyGen (China). Enhanced ATP assay kits and cell-lysis RIPA buffer were purchased from the Beyotime Institute of Biotechnology. Antibodies, such as STAT3, p-STAT3(Y705), RAD51, γ-H_2AX, Cyclin B1, LC3B, p62, MMP9, COX2, GAPDH, and Tubulin, for western blot or immunofluorescence, were purchased from the Proteintech. Deuterated solvents for NMR purposes were obtained from Merck and Cambridge Isotopes. Other organic reagents, which were of analytical grade, were obtained from domestic chemical corporations and used as received without any further purification.

The ^1H and ^{13}C NMR spectra were recorded on a Bruker AVANCE 400 spectrometer at ambient temperature. Electrospray ionization mass spectra (ESI-MS) were obtained using an LCQ spectrometer (Thermo Scientific). UV-Vis absorption spectra was recorded on a Lambda 365 UV-Vis spectrophotometer. The Cells used in this work were incubated in a humidified incubator (BB 150 CO_2 incubator, Thermo Fisher Scientific, USA). MTT data were recorded on a microplate reader (LabServ K3, Part of Thermo Fisher Scientific, USA). Iridium contents in the samples were determined by ICP-MS (X Series 2, Thermo Fisher, USA). The confocal imaging was performed on a confocal laser scanning microscope (Nikon A1, Japan). Flow cytometric analysis was done using a flow cytometer (BD FACSVerse, USA). Western blotting experiments were conducted on Tanon 5200 Multi and the signals were enhanced by Tanon High-sig ECL Western Blotting Substrate.

Synthesis of ligand and complex

Synthesis of Ligand py-ART

ART (0.384 g, 1 mmol), 4-pyridylcarbinol (0.109 g, 1 mmol) and DMAP (0.132 g, 1.08 mmol) were dissolved in anhydrous DMF (15 mL) and stirred in an ice bath for 30 min. Then EDCI (0.207 g, 2 mmol) was added to the reaction mixture and stirred at room temperature for 24 h. After the reaction was completed, the DMF solvent was removed under pressure, and the compound was purified by column chromatography using 10% methanol in dichloromethane as eluent. Finally, 0.219 g pure product was obtained with a yield of 46%. ^{1}H NMR (400 MHz, DMSO-d_6) δ 8.56 (d, J = 5.0 Hz, 2H), 7.34 (d, J = 5.1 Hz, 2H), 5.67 (d, J = 9.7 Hz, 1H), 5.56 (s, 1H), 5.16 (s, 2H), 2.73 (s, 4H), 2.28 (s, 1H), 2.17 (t, J = 13.9 Hz, 1H), 1.99 (d, J = 13.8 Hz, 1H), 1.81 (d, J = 7.3 Hz, 1H), 1.60 (d, J = 9.5 Hz, 2H), 1.53 (s, 1H), 1.50 – 1.33 (m, 3H), 1.27 (s, 4H), 1.18 (dd, J = 11.3, 6.4 Hz, 1H), 0.88 (d, J = 6.3 Hz, 3H), 0.72 (d, J = 7.1 Hz, 3H). ^{13}C NMR (101 MHz, DMSO) δ 172.07, 171.34, 150.13, 145.58, 122.16, 104.04, 92.33, 91.07, 80.32, 64.30, 51.57, 45.01, 36.41, 36.34, 34.16, 32.08, 29.06, 28.84, 25.97, 24.65, 21.44, 20.52, 12.15.

Synthesis of complexes (Ir-ART and Ir-OH)

The iridium precursor Ir(Cp*)(ppy)Cl was synthesized and purified as previously reported.[1] The complexes **Ir-ART** and **Ir-OH** were prepared by the similar method following the reference.[2] Generally, a solution of the chloride complex Ir(Cp*)(ppy)Cl (0.1 mmol, 0.052 g) and $AgNO_3$ (1 mmol) in MeOH/H_2O (1:1, v/v) was refluxed in the dark under the argon atmosphere for 3 h. Then, the precipitate (AgCl) was removed by filtration, and the ligand py-ART or 4-pyridylcarbinol (1 mmol) was added to the filtrate. The reaction mixture was refluxed under the argon atmosphere overnight, which were then exchanged with NH_4PF_6 (10 mmol).

Ir-ART. The precipitate was collected and purified by column chromatography on silica gel (dichloromethane/methanol = 20/1) to give pure product. Complex **Ir-ART** was obtained as a yellow powder. Yield: 0.046 g (47 %). ^{1}H NMR (400 MHz, $CDCl_3$) δ 9.23 (d, J = 5.7 Hz, 1H), 8.52 (d, J = 5.8 Hz, 2H), 7.89 (d, J = 7.6 Hz, 1H), 7.86 – 7.77 (m, 2H), 7.64 (t, J = 6.7 Hz, 2H), 7.38 (t, J = 7.6 Hz, 1H), 7.28 (s, 1H), 7.23 – 7.16 (m, 1H), 5.75 (d, J = 10.0 Hz, 1H), 5.43 (s, 1H), 5.09 (s, 2H), 2.71 (d, J = 6.4 Hz, 4H), 2.53 (s, 1H), 2.38 (s, 1H), 2.25 (s, 3H), 2.04 (d, J = 14.3 Hz, 1H), 1.91 (s, 1H), 1.80 – 1.70 (m, 3H), 1.63 (s, 15H), 1.41 (d, J = 5.5 Hz, 3H), 1.27 (s, 3H), 0.98 (s, 3H), 0.81 (s, 3H). ^{13}C NMR (101 MHz, $CDCl_3$) δ 171.51, 171.46, 171.01, 170.99, 166.76, 160.84, 153.37, 152.40, 148.31, 148.28, 145.36, 139.05, 134.75, 131.99, 131.95, 125.29, 124.64, 124.61, 124.40, 123.93, 123.90, 119.21, 104.48, 92.34, 91.49, 90.38, 80.11, 63.24, 51.53, 45.18, 37.29, 36.20, 34.06, 31.76, 29.70, 28.97, 28.56, 25.93, 24.58, 21.97,

20.22, 12.02, 8.61. HR-MS (CH_3OH) calcd for: [**Ir-ART**-PF_6^-]$^+$ m/z = 957.3666, found m/z = 957.3641.

Ir-OH. Pure product of **Ir-OH** was obtained by recrystallizing from dichloromethane/diethyl ether. Complex **Ir-OH** was obtained as a yellow powder. Yield: 0.029 g (49 %). ^{1}H NMR (400 MHz, $CDCl_3$) δ 9.04 (s, 1H), 8.31 (s, 2H), 7.91 (d, J = 7.5 Hz, 1H), 7.82 (d, J = 8.3 Hz, 2H), 7.65 (d, J = 7.6 Hz, 1H), 7.54 (s, 1H), 7.40 (s, 1H), 7.28 (s, 2H), 7.19 (s, 1H), 4.62 (s, 2H), 1.62 (s, 15H). ^{13}C NMR (101 MHz, $CDCl_3$) δ 167.34, 160.89, 156.09, 152.01, 151.61, 151.09, 149.15, 144.95, 139.05, 134.56, 132.22, 124.59, 124.42, 124.00, 121.39, 119.64, 90.17, 62.76, 61.69, 29.69, 8.56. ESI-MS (CH_3OH) calcd for: [**Ir-OH**-PF_6^-]$^+$ m/z = 591.2, found m/z = 591.2.

Stability studies

The stock solutions (10 mM) of **Ir-ART** were prepared with DMSO and stored in a 4 °C refrigerator. Complexes **Ir-ART** (20 μM) were dissolved in 1 mL of freshly prepared DMSO/PBS (v/v, 1/99) solution, and then a suspension of PLE (0.2 U/mL) in saturated $(NH_4)_2SO_4$ solution was added. After the mixtures were incubated at 37 °C for the indicated time intervals (0, 24 h), acetone (400 μL) was added to quench the enzymatic effect. The samples were centrifuged (15000 g, 10 min). The supernatants were collected and analyzed by ESI-MS.

Lipophilicity

According to the reported procedure,[3] the lipophilicity (Log$P_{o/w}$) of **Ir-ART** and **Ir-OH** was measured by the flask-shaking method.

Cell lines and culture conditions

HCT-116, A549, HeLa, and MCF-7 cells were obtained from the Experimental Animal Center of Sun Yat-Sen University (Guangzhou, China). Cells were routinely cultured in DMEM (Dulbecco's modified Eagle's medium, Gibco BRL) or RPMI 1640 (Roswell Park Memorial Institute 1640, Gibco BRL) medium containing 10 % FBS (fetal bovine serum, Gibco BRL), 100 μg/mL streptomycin, and 100 U/mL penicillin (Gibco BRL). The cells were cultured in tissue culture flasks in a humidified incubator (BB 150 CO_2 incubator, Thermo Fisher Scientific, USA), which provided an atmosphere of 5% CO_2 and 95% air at a constant temperature of 37 °C. In each experiment, cells treated with the vehicle DMSO (1%, v/v) were used as the control group.

Cytotoxicity

The cytotoxicity of the complexes toward cancer cells were determined via MTT assays. Cells (100 μL) were seeded to a 96-well plate at a density of 5 × 10^4 cells/mL. After 24-hour incubation, cells were incubated with different concentrations of complexes and incubated for 48 h at 37 °C. Then an amount of 20 μL MTT (5 mg/mL, PBS buffer) was added to each well and incubated for another 4 h. The medium was carefully removed and dissolved in DMSO (150 μL per well) was added and the absorbance at 570 nm of the purple formazan was measured by microplate reader (LabServ K3, Thermo Scientific). Each well was repeated three times to obtain the mean value. The IC_{50} values quoted are mean ± standard deviation. The maximum DMSO concentration in all cases was controlled at 1% (v/v).

ICP-MS measurement

The cellular uptake and distribution of **Ir-ART** and **Ir-OH** in HCT-116 cells were determined by measuring the iridium contents. Briefly, cells were seeded and incubated overnight under standard growth conditions. And then incubated with the complexes **Ir-ART** (10 μM) and **Ir-OH** (10 μM) for 6 h. Then the cells were washed with PBS, collected by trypsin, stored in tubes, and resuspended in PBS. Mitochondria and nuclei were separated from the pellets using cell mitochondria isolation kit (KeyGen, China). These fractions in the stock buffer were then digested with concentrated nitric acid (100 μL) at 95 °C for 2 h, hydrogen peroxide (30 %, 50 μL) at 95 °C for 1.5 h, and concentrated hydrochloric acid (50 μL) at 95 °C for 1.5 h to give fully homogenized solutions. The remaining solutions were diluted to 2 mL with doubly distilled water and iridium content in the samples was determined by ICP-MS (X Series 2, Thermo Fisher, USA). The average of three parallel experimental data was reported as the final results.

ROS detection

The ROS generation in HCT-116 cells was measured by DCFH-DA staining. HCT-116 cells were seeded in a 6-well plate at a density of 2 × 10^5 cells/well and cultured for 12 h at 37 °C. After that, complexes were added at the indicated concentrations. After 24 h incubation, DCFH-DA was added and incubated for another 30 min. Then the cells were washed with fresh medium three times and analyzed using a BD FACS Calibur flow cytometer with excitation at 488 nm and emission at 520 ± 20 nm. The data was analyzed using FlowJo 7.6.1 software. 10, 000 cells were acquired for each sample. For the confocal microscopy analysis of ROS, HCT-116 cells were seeded into 35 mm confocal dishes (JET

BIOFIL, Canada) for 12 h and incubated with the indicated complexes for 24 h. Subsequently, the medium was replaced with the ROS probe DCFH-DA and washed with fresh medium three times. Then the cells were viewed im-mediately under a confocal microscope (A1, Nikon).

Mitochondrial membrane potential detection

The MMP of A549 cells was measured by JC-1 assay kit. HCT-116 cells were seeded in a 6-well plate at a density of 2×10^5 cells/well and cultured for 12 h at 37 °C. After that, complexes were added at the indicated concentrations. After 24 h incubation, the cells were harvested with 0.25% trypsin and washed with PBS three times. After the addition of 0.5 mL JC-1 working solution, the cells were incubated at 37°C for 30 min. Then the staining solution was removed, and the cell samples were washed with 1× incubation buffer and analyzed by flow cytometry using a BD FACS Calibur flow cytometer. The excitation wavelength was 488 nm, and the emission band path for the green channel was 520±20 nm, while that for the red channel was 580±20 nm. For the confocal microscopy analysis of MMP, HCT-116 cells were seeded into 35 mm confocal dishes for confocal microscopy. After being cultured overnight, the cells were treated with the indicated complexes for 24 h at 37 °C. After being stained with a JC-1 working solution for 30 min, the cells were washed with 1× incubation buffer and then immediately observed by confocal microscopy (A1, Nikon, Japan).

Cell cycle analysis

HCT-116 cells were seeded in 6-well cell plates and **Ir-ART** with desired concentrations was added after incubation at 37 °C overnight in an atmosphere of 5% CO_2 and 95% air. After treatment with **Ir-ART** for 24 h at 37 °C, cells were collected by centrifugation and washed twice with PBS. Then cells were fixed with 70% ethanol overnight at 4 °C. The fixed mixture was washed twice with cold PBS, incubated with 500 μL propidium iodide (PI) for 30 min after pretreatment with RNase A (100 μg/mL) for 10 min, washed with PBS twice, and then the cell samples were detected on flow cytometry and analyzed a BD FACS Calibur flow cytometer.

Intracellular ATP detection assay

HCT-116 cells were cultured in a 6-well plate with a density of 2×10^5 cells/well and incubated overnight at 37 °C. Then the cells were treated with the indicated complexes for 24 h. The content of intracellular ATP was measured by ATP assay kit according to the manufacturer's protocol.

Wound healing assay

HCT-116 cells were seeded in 6-well plates at 20 × 10^4 cells per well. The cells were allowed to adhere to the surface, and once they reached 90% confluence, the monolayer was wounded using a 200-μl pipette tip to streak each hole vertically. The cells were washed with PBS three times and incubated in serum-free RPMI 1640 containing the drugs for another 24 h. Images of the scratches were captured with a microscope camera. Data were collected from three independent experiments.

Transwell migration assay

Transwell chambers with 8-μm pores (Corning, NY) were used to evaluate the invasion of HCT-116 cells. The upper chamber was filled with 1 × 10^5 cells from each sample in serum-free medium for invasion analysis. After incubating the cells at 37 °C for 24 h in medium containing the drugs, the cells migrating to the bottom of the filter were incubated with 0.5% crystal violet at room temperature for 10 min. For quantitative measurement, five areas (at ×100 magnification) were randomly selected under an optical microscope, and the cells were counted.

Western blot assay

HCT-116 cells were cultured in 10 cm dishes and separately treated with indicated complexes for 24 h at 37 °C. The cells were harvested and lysed in cell lysis buffer containing PMSF (1%) for 30 min on ice. The lysates were collected by centrifugation at 13000 rpm at 4 °C for 20 minutes. Proteins from cell lysates were separated on SDS-PAGE and transferred to polyvinylidine difluoride (PVDF) membrane (Millipore). The membrane was blocked with PBST containing 5% skimmed milk powder for 1 hour, and further incubated with specific antibodies at 4 °C overnight with gentle shaking. After that, the membrane was incubated with the secondary antibody at room temperature for 1 hour. Protein blots were detected by chemiluminescence reagent (Tanon).

Immunofluorescence assay

HCT-116 cells were seeded into 35 mm confocal dishes (JET BIOFIL, Canada) for confocal microscopy. After cultured overnight, the cells were treated with indicated complexes for 24 h at 37 °C. Then HCT-116 cells were fixed with 4% paraformaldehyde/PBS for 30 min at the room temperature. After washed with PBS for three times, cells were then stained with anti-LC3B antibodies for 1 h at room temperature, and detected with a secondary antibody Fluorescein (FITC)-conjugated Affinipure goat anti-

rabbit IgG. The cells were counterstained with 300 nM DAPI for 10 min and immediately observed by confocal microscopy.

Detection of PGE2

HCT-116 cells were seeded in 6-well plates at a density of 2×10^5 cells per well and cultivated for 24 h. Then, cells treated with indicated complexes for 24 h, respectively. The amount of secreted PGE2 in the cell culture cytoplasm was determined using a PGE2 ELISA kit according to the manufacturer's protocol.

Statistical analysis

Data are given as mean ± standard deviation (SD). Statistical significance was performed using an unpaired two-tailed Student's *t*-test or one-way analysis of variance (ANOVA) with Bonferroni's post-test. Statistical significance was set at $^{*}P < 0.05$, and extreme significance was set at $^{**}P < 0.01$, $^{***}P < 0.001$ and $****P < 0.0001$.

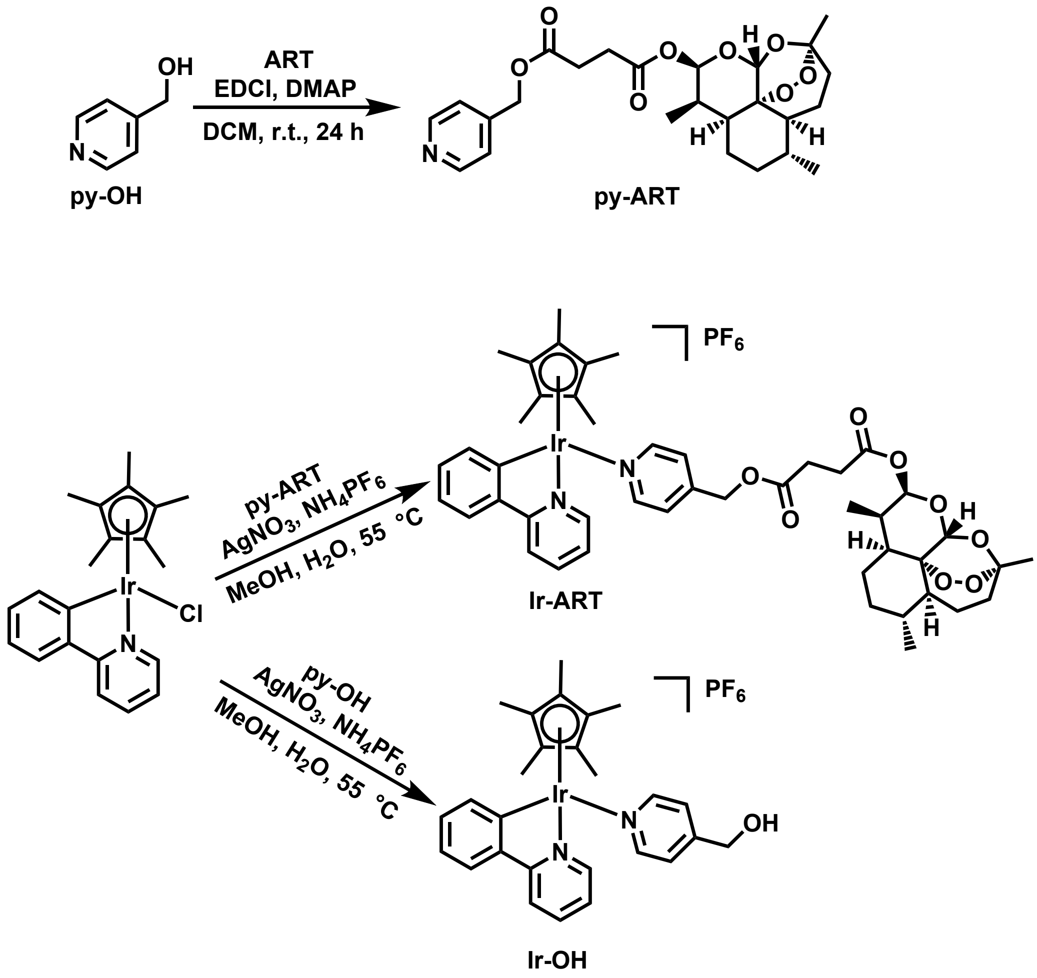

Scheme S1. Synthetic routine of the py-ART and complex **Ir-ART**, **Ir-OH**.

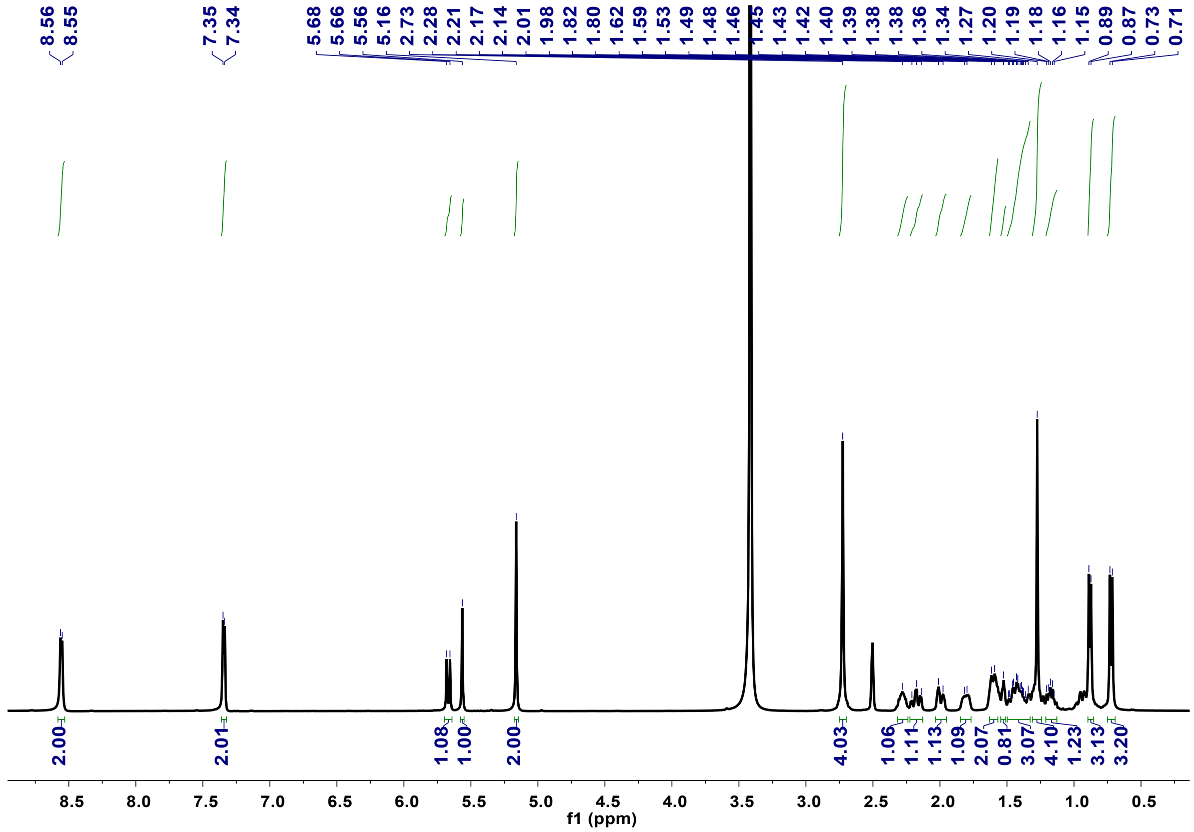

Fig. S1. ^{1}H NMR spectrum (400 MHz, d_6-DMSO) of py-ART.

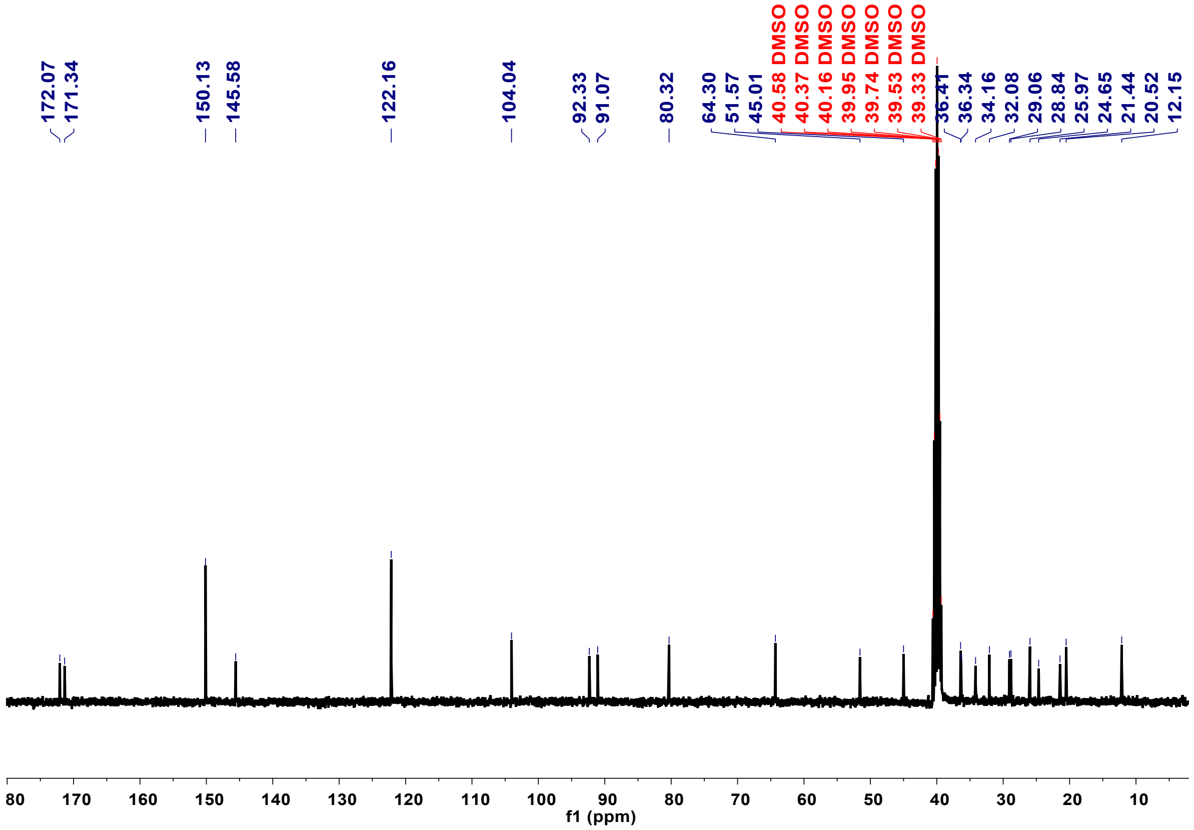

Fig. S2. ^{13}C NMR spectrum (101 MHz, d_6-DMSO) of py-ART.

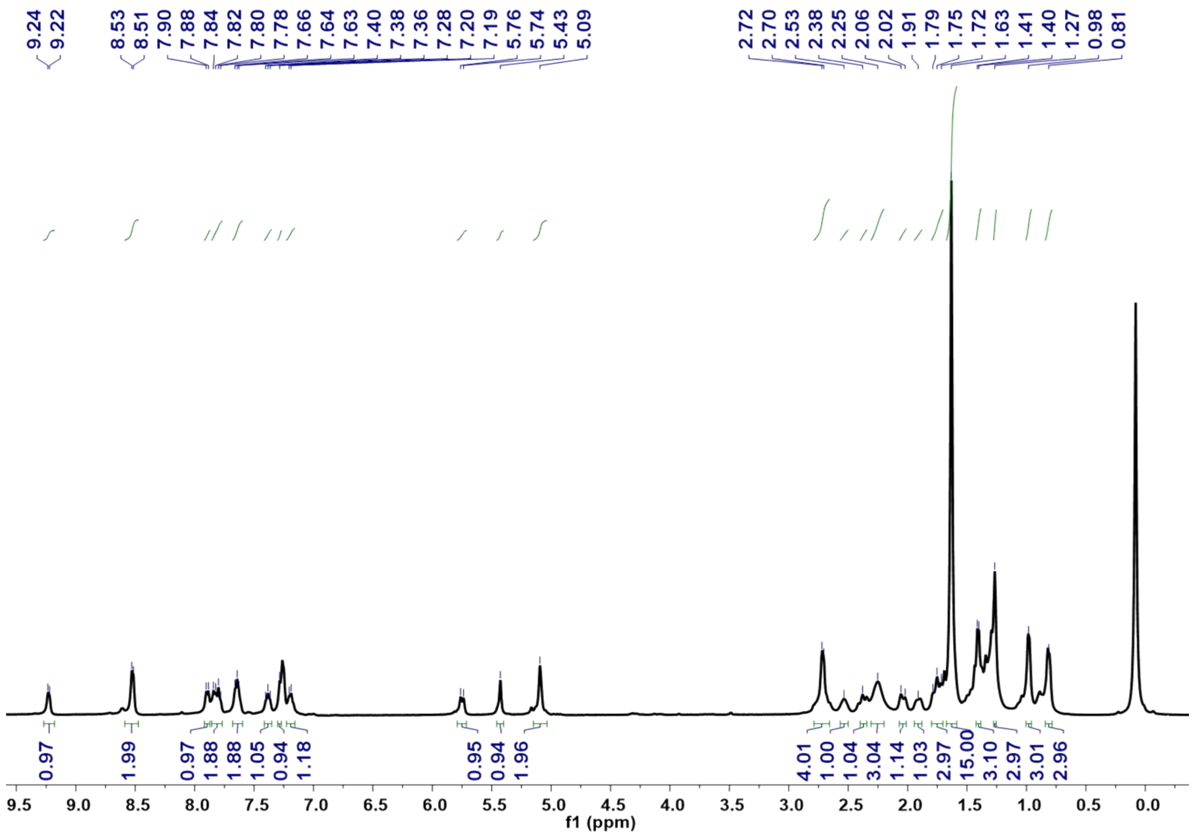

Fig. S3. ^{1}H NMR spectrum (400 MHz, $CDCl_3$) of complex **Ir-ART**.

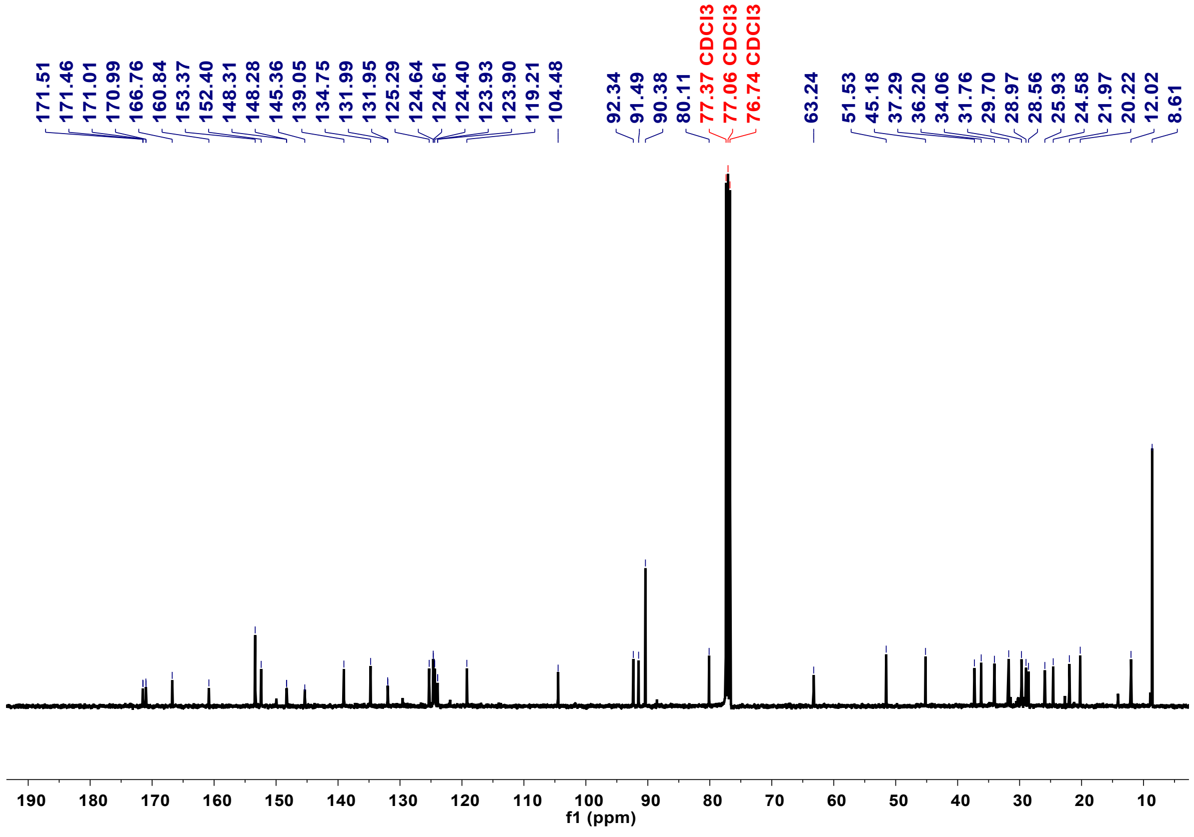

Fig. S4. ^{13}C NMR spectrum (101 MHz, $CDCl_3$) of complex **Ir-ART**.

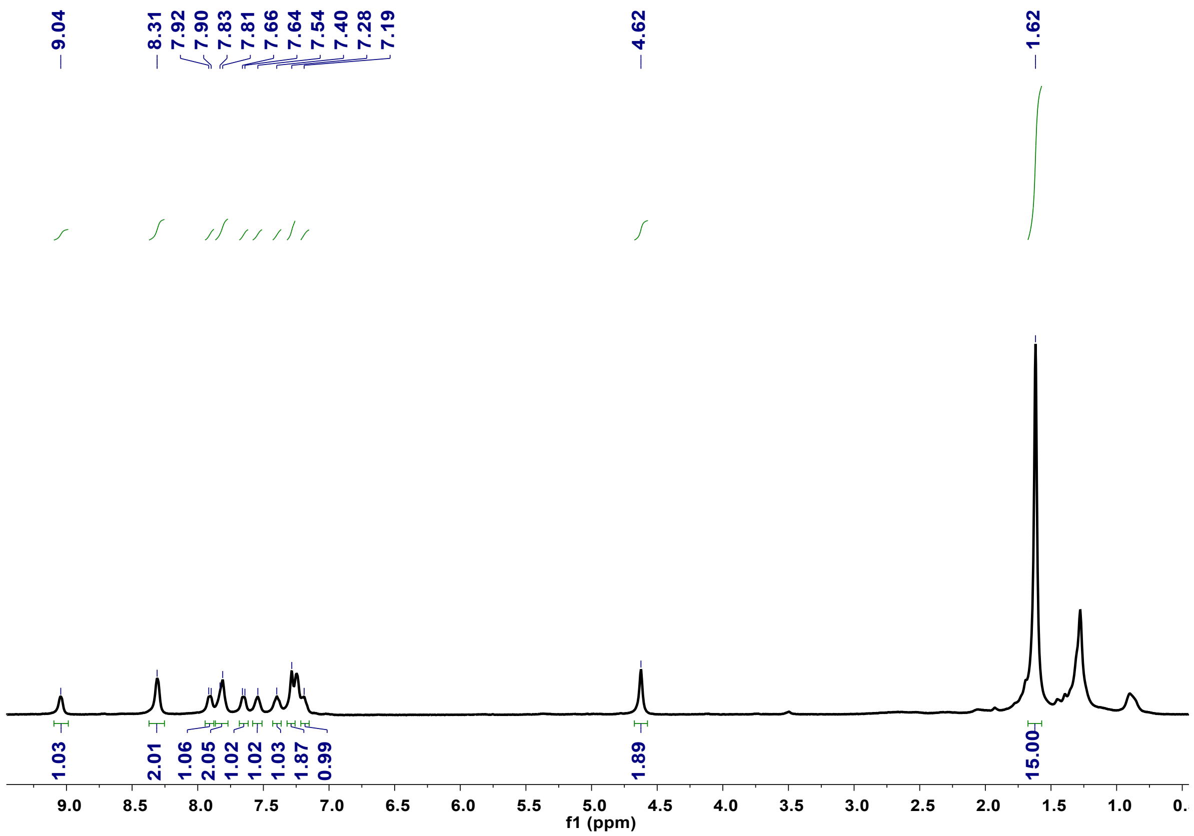

Fig. S5. ^{1}H NMR spectrum (400 MHz, $CDCl_3$) of complex **Ir-OH**.

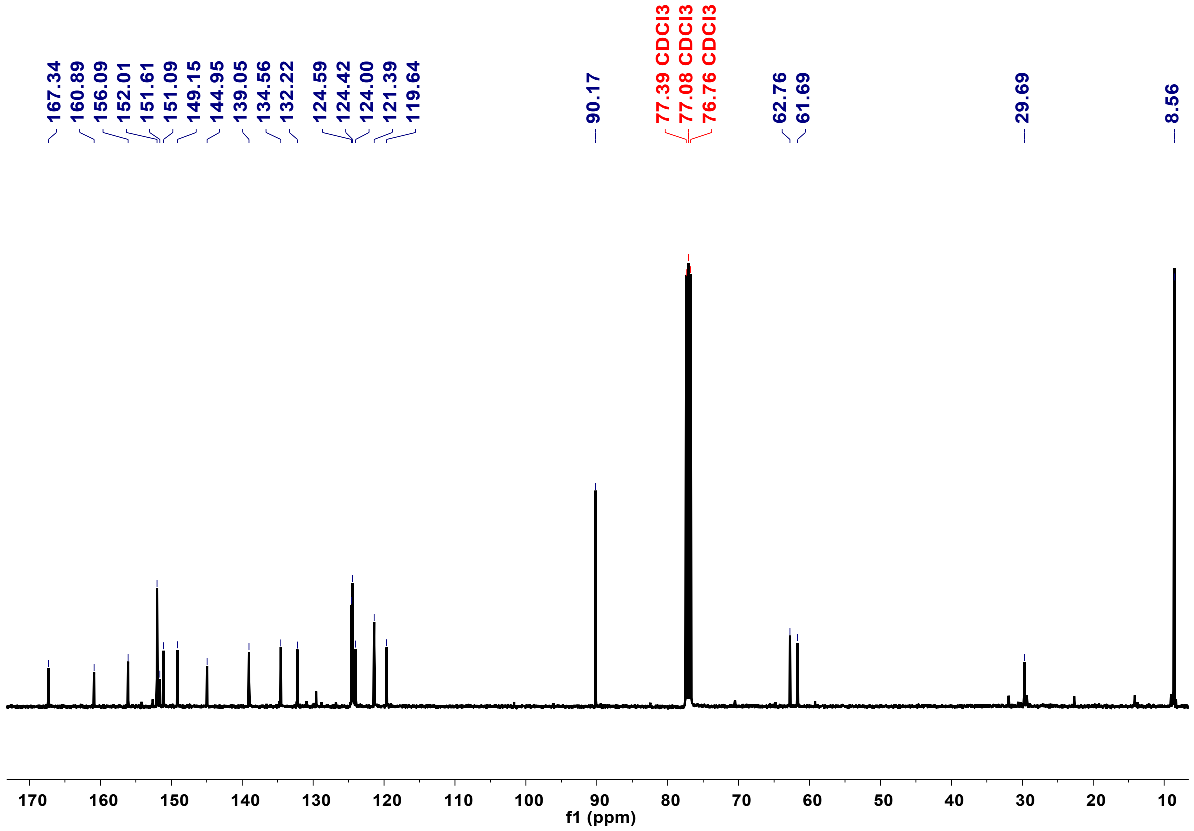

Fig. S6. ^{13}C NMR spectrum (101 MHz, $CDCl_3$) of complex **Ir-OH**.

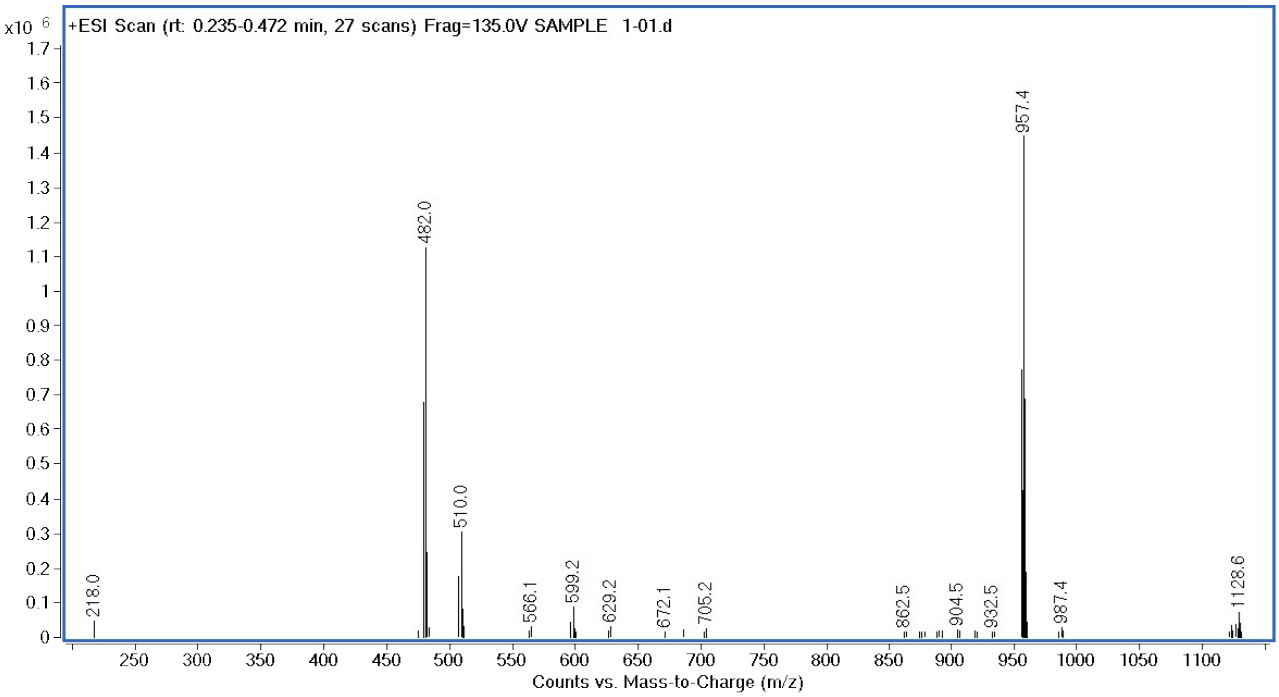

Fig. S7. ESI-MS spectrum (CH_3OH) of complex **Ir-ART**.

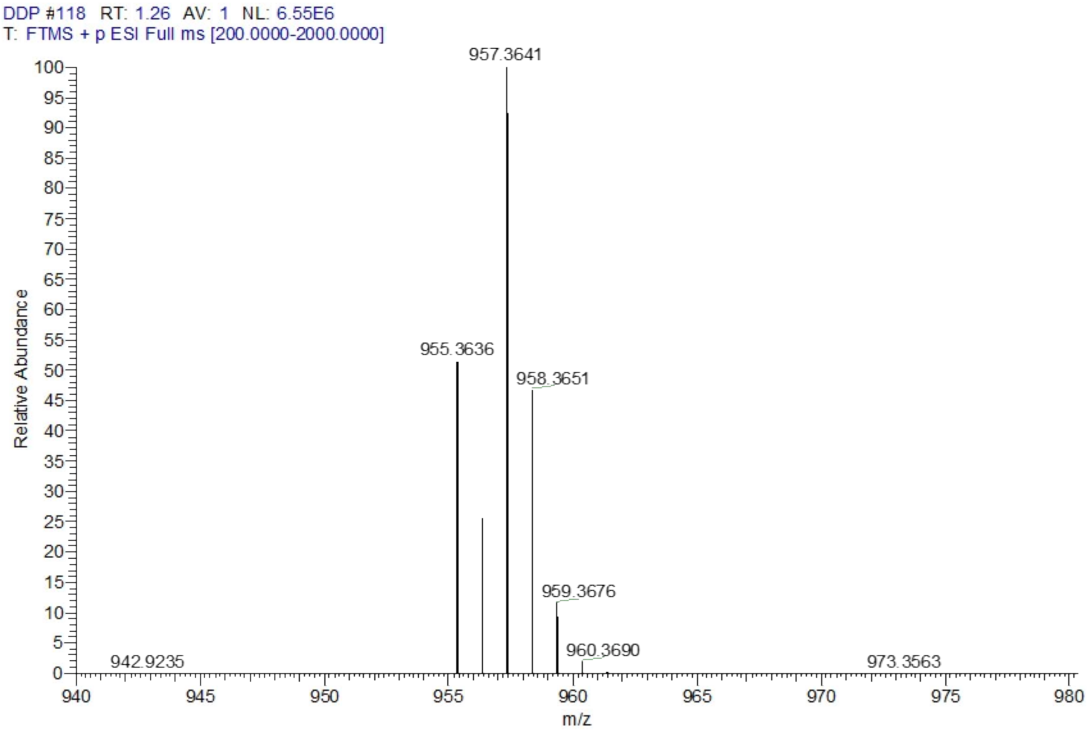

Fig. S8. HR-MS spectrum (CH_3OH) of complex **Ir-ART**.

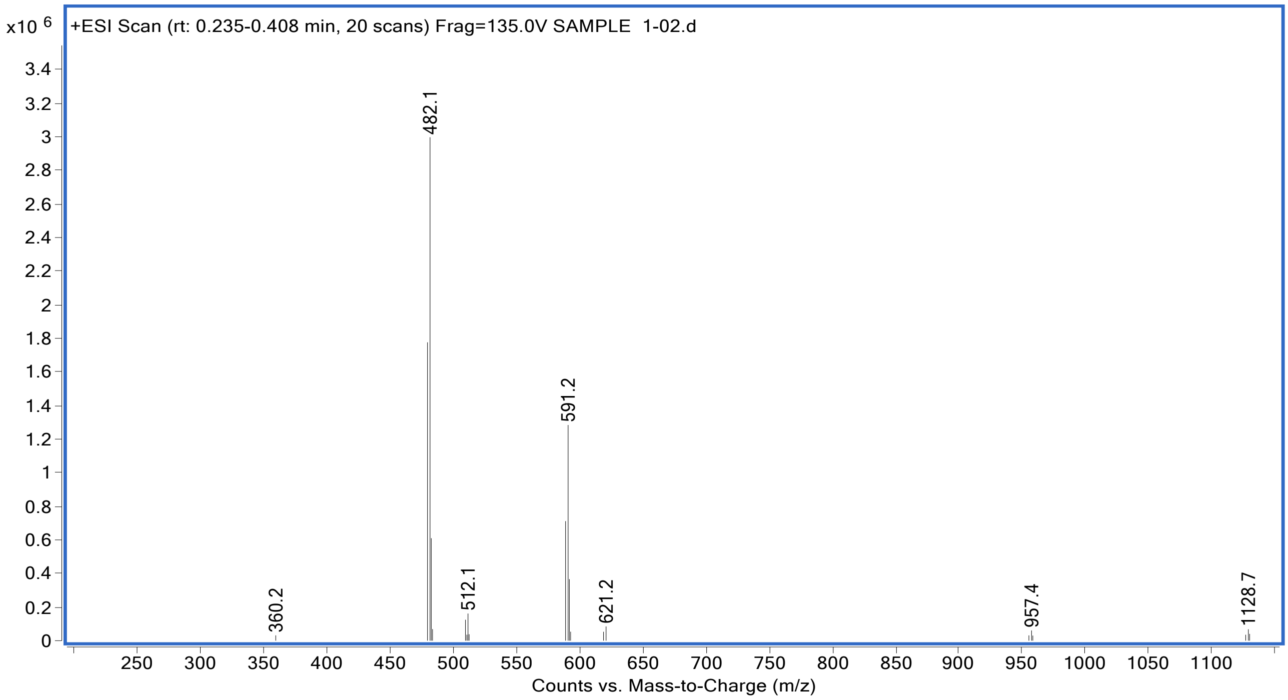

Fig. S9. ESI-MS spectrum (CH_3OH) of complex **Ir-OH**.

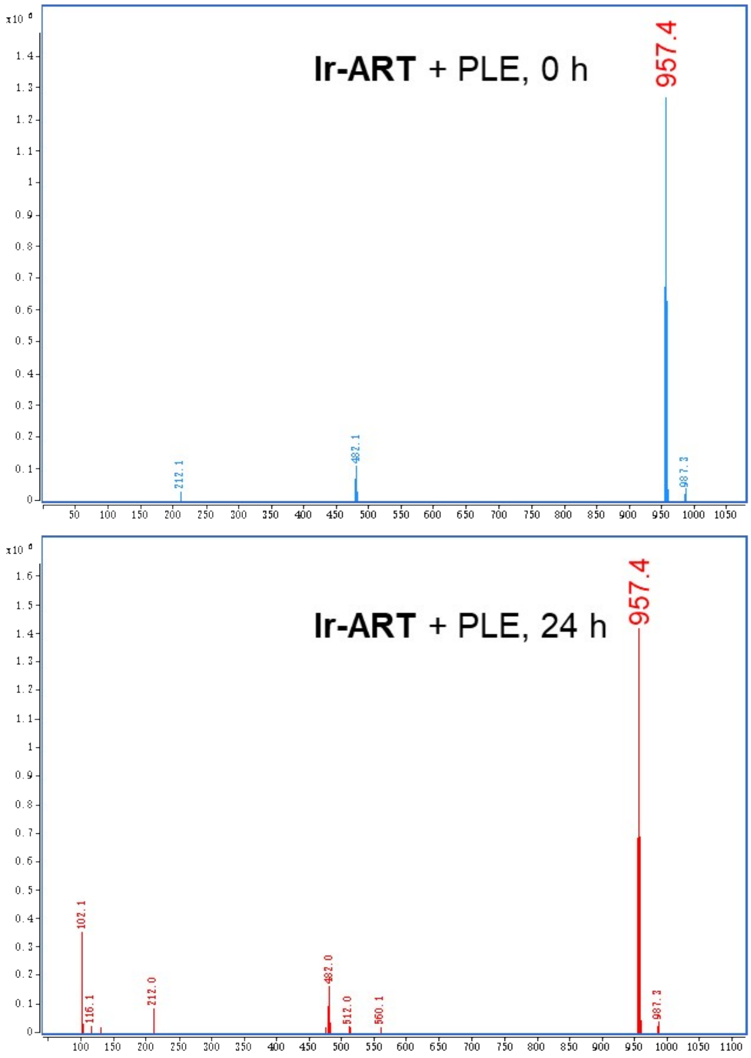

Fig. S10. ESI-MS spectra of **Ir-ART** dispersed in PBS solution upon treatment with porcine liver esterase (PLE, 0.2 U/mL) at 37 °C for 0 and 24 h.

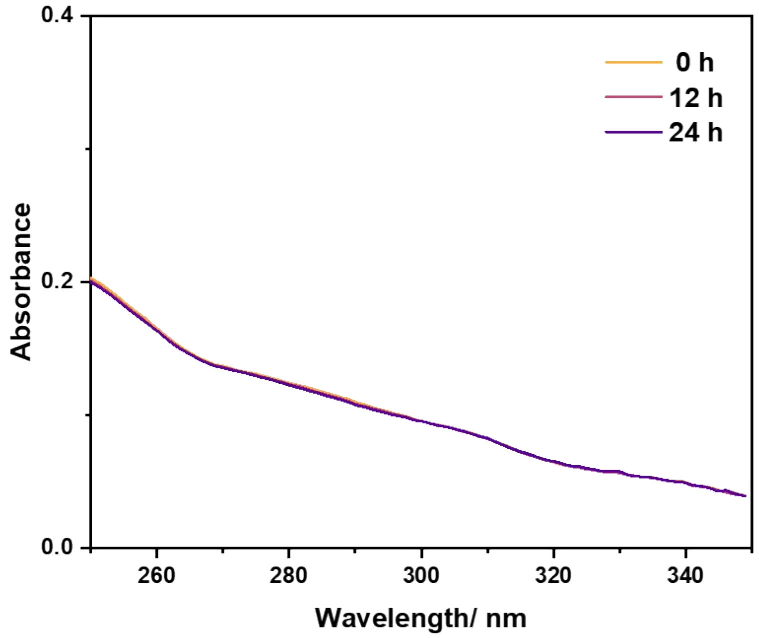

Fig. S11. UV-vis spectra for **Ir-ART** (10 μM) in DMSO/DMEM culture medium (v/v, 1/99) solution was recorded for a period of 24 h at 37 °C.

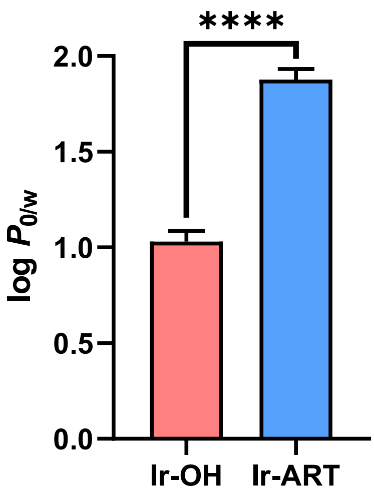

Fig. S12. The lipophilicity (log $P_{o/w}$) of **Ir-OH** and **Ir-ART** determined by the saturation shake-flask method.

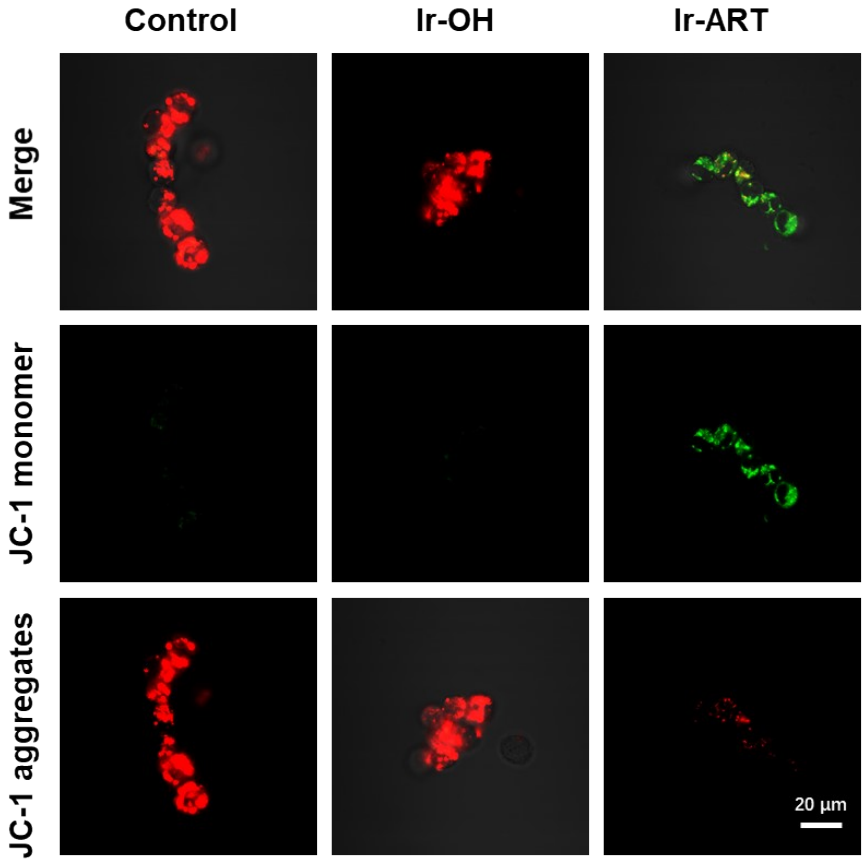

Fig. S13. Confocal images of JC-1-labeled HCT-116 cells treated with **Ir-OH** and **Ir-ART** (10 μM).

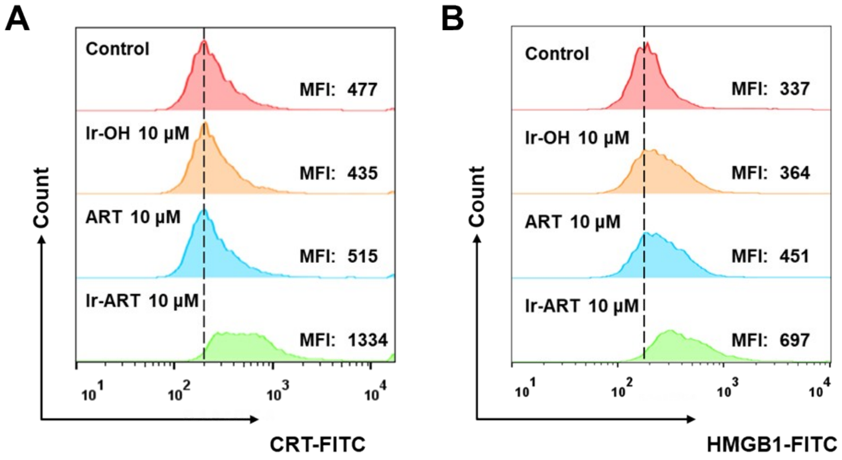

Fig. S14. Flow cytometric analysis of the expression of CRT and HMGB1 in HCT-116 cells treated with indicated complexes.

References

1. Z. Liu, A. Habtemariam, A. M. Pizarro, G. J. Clarkson and P. J. Sadler, *Organometallics*, 2011, **30**, 4702-4710.
2. M.-M. Wang, D.-P. Deng, A.-M. Zhou, Y. Su, Z.-H. Yu, H. K. Liu and Z. Su, *Inorg. Chem.*, 2024, **63**, 4758-4769.
3. J. Yang, M.-M. Wang, D.-P. Deng, H. Lin, Y. Su, C.-X. Shao, S.-H. Li, Z.-H. Yu, H.-K. Liu and Z. Su, *Inorg. Chem.*, 2024, **63**, 5235-5245.
